# Phase Separation, Material State, and Condensate Fate in Mammalian Autophagy

**DOI:** 10.1002/advs.77360

**Published:** 2026-09-01

**Authors:** Yuanqiang Lin, Mingran Dai, Enyong Dai, Rui Kang, Daniel J. Klionsky, Daolin Tang, Yu Sun, Ningning Lv, Junnan Jiang

**Affiliations:** ^1^ Department of Ultrasound China‐Japan Union Hospital of Jilin University Changchun Jilin China; ^2^ Engineering Research Center of the Chinese Ministry of Education for Bioreactor and Pharmaceutical Development College of Life Sciences Jilin Agricultural University Changchun Jilin China; ^3^ Second Ward of the Oncology Department China‐Japan Union Hospital of Jilin University Changchun Jilin China; ^4^ Department of Surgery UT Southwestern Medical Center Dallas Texas USA; ^5^ Life Sciences Institute and Department of Molecular Cellular and Developmental Biology University of Michigan Ann Arbor Michigan USA; ^6^ Department of Interventional Radiology Oncology Center China‐Japan Union Hospital of Jilin University Changchun Jilin China; ^7^ Department of Gastrointestinal and Colorectal Surgery China‐Japan Union Hospital of Jilin University Changchun Jilin China

**Keywords:** autophagy, condensate, disease, phase separation, receptor

## Abstract

Biomolecular phase separation has emerged as a key organizing principle in macroautophagy (hereafter autophagy). In mammalian cells, phase‐separated condensates not only serve as substrates for selective degradation, but also act as dynamic platforms for cargo recognition, signaling integration, and autophagosome assembly. The material state of these condensates is an important determinant of autophagic fate. Condensates exist along a continuum ranging from liquid‐like droplets to gel‐like and solid assemblies, and their progressive maturation can alter accessibility to autophagic machinery. Scaffold proteins and selective autophagy receptors further organize these assemblies into degradation‐competent mesoscale reaction fields that couple cargo recognition with phagophore formation. Dysregulation of this phase separation–autophagy axis is increasingly implicated in neurodegeneration, cancer, aging, and stress‐associated degenerative disease. Here, we propose a multiscale framework in which molecular accessibility, mesoscale organization, and condensate state transitions collectively shape autophagic outcome, providing a conceptual basis for predictive models and therapeutic strategies aimed at restoring condensate degradability.

**Abbreviations**
ATP: adenosine triphosphate
ER: endoplasmic reticulum
FRAP: fluorescence recovery after photobleaching
IDRs: intrinsically disordered regions
LIR: LC3‐interacting region
LLPS: liquid–liquid phase separation
MTORC1: mechanistic target of rapamycin kinase complex 1
SNARE: soluble *N*‐ethylmaleimide‐sensitive factor attachment protein receptor protein

## 1 Introduction

Macroautophagy (hereafter autophagy) is a lysosome‐dependent pathway that maintains cellular homeostasis by engulfing and degrading cytoplasmic material, including damaged organelles, protein aggregates, and other aberrant macromolecular assemblies [1]. In mammalian cells, selective autophagy is particularly important for proteostasis, as it couples cargo recognition to the recruitment of core autophagic machinery through dedicated receptors [2]. Failure of this surveillance system leads to the accumulation of abnormal protein assemblies and contributes to the pathogenesis of neurodegenerative disease, cancer, metabolic dysfunction, and age‐associated proteotoxic stress [3].

Over the past decade, biomolecular condensates have emerged as a fundamental organizing principle of cellular architecture. Frequently formed through or associated with liquid–liquid phase separation (LLPS), these membraneless assemblies arise from multivalent interactions among proteins and nucleic acids and occupy a continuum of material states ranging from highly dynamic liquid‐like droplets to viscoelastic gels and solid‐like assemblies [4]. Rather than serving as passive storage compartments, condensates organize biochemical reactions, coordinate stress responses, regulate molecular exchange, and dynamically remodel intracellular organization according to cellular demands [4].

Autophagy is now increasingly viewed within this condensate‐centered framework of cell organization. Beyond pioneering studies in yeast, *Caenorhabditis elegans* (*C. elegans*), and plants [5, 6, 7, 8, 9], accumulating evidence demonstrates that phase separation contributes to multiple steps of mammalian autophagy, including autophagosome initiation, cargo clustering, receptor‐mediated signaling, and regulation of stress‐responsive ribonucleoprotein assemblies [10, 11]. These findings suggest that condensates are not merely substrates of selective autophagy but actively participate in determining cargo recognition, phagophore assembly, and degradative outcome.

A central unresolved question, however, is why some condensates are efficiently recognized and eliminated whereas others progressively mature into persistent assemblies that resist autophagic clearance. Although condensate material state is increasingly recognized as an important determinant, degradative outcome is likely governed by the coordinated interplay of condensate biophysics, receptor accessibility, mesoscale organization, membrane interactions, and lysosomal competence. Understanding how these factors collectively determine condensate fate has become a major challenge in mammalian selective autophagy.

Several excellent reviews have summarized the roles of phase separation in autophagy or the biophysical principles governing condensate formation [10, 11]. Here, rather than focusing on individual molecular mechanisms, we ask a different conceptual question: how is condensate state translated into distinct autophagic outcomes in mammalian cells? To address this question, we introduce condensate degradability as a systems‐level concept describing the propensity of condensates to undergo productive autophagic recognition, sequestration, and degradation. Building upon recent advances, we propose a multiscale framework that integrates condensate biophysics, mesoscale organization, receptor accessibility, membrane engagement, and lysosomal competence into a unified model linking condensate state to autophagic fate (Figure 1). Within this framework, condensate material state is viewed as an important, but not sufficient, determinant of degradation, whose effects emerge through interactions with higher‐order organizational and cellular factors.

**FIGURE 1:**
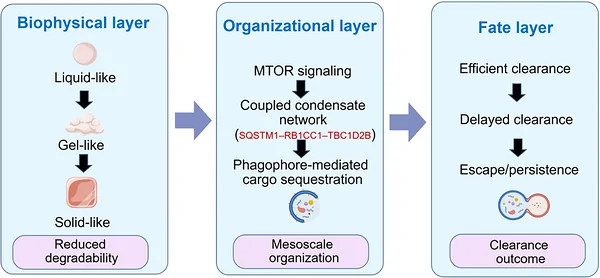
Multiscale framework linking condensate material properties, mesoscale organization, membrane wetting, and autophagic outcomes. Biomolecular condensates exhibit a continuum of material states, transitioning from liquid‐like to gel‐like and solid‐like assemblies, with progressively reduced degradability. At the organizational level, nutrient and stress cues converge on MTOR signaling to regulate a coupled condensate network comprising SQSTM1, RB1CC1, and TBC1D2B. Within this mesoscale system, SQSTM1 condensates concentrate ubiquitinated cargo, whereas RB1CC1 and TBC1D2B condensates coordinate autophagy initiation and early phagophore assembly. Condensate material state is an important, but not sufficient, determinant of autophagic fate. Instead, condensate degradability emerges from the combined effects of material properties, molecular accessibility, receptor engagement, mesoscale organization, membrane compatibility, and lysosomal competence. Within this working model, these interconnected factors are proposed to collectively influence phagophore engagement and cargo sequestration, thereby biasing condensates toward efficient clearance, delayed degradation, or pathological persistence. Additional variables, including membrane curvature, receptor density, MAP1LC3‐LIR interactions, local lipid composition, and lysosomal capacity, may further modulate these outcomes. The framework further defines representative measurable biophysical and cellular parameters (summarized in Table 1) that can be integrated into predictive and experimentally testable models of condensate fate across multiple biological scales.

Importantly, this framework is intended to be conceptually rigorous and experimentally testable. Accordingly, Table 1 summarizes representative measurable parameters, corresponding experimental approaches, and their predicted relationships with condensate degradability, providing practical criteria for evaluating and refining the proposed framework. Because not all condensate‐associated structures satisfy the stringent criteria for LLPS, and recruitment of autophagy‐related proteins does not necessarily indicate productive degradation, we also distinguish experimentally validated LLPS from condensate‐like assemblies throughout this review. Table 2 summarizes the strength and source of evidence supporting representative condensate‐autophagy mechanisms, thereby defining the methodological boundaries of the framework and distinguishing well‐established mechanisms from emerging concepts.

**TABLE 1:** Representative measurable parameters underlying the predictive condensate‐fate framework.

Framework layer	Representative measurable parameter	Representative experimental approach	Predicted implication for condensate fate
Biophysical state	FRAP recovery (molecular exchange)	Fluorescence recovery after photobleaching (FRAP)	High recovery predicts efficient clearance, whereas reduced recovery predicts delayed turnover or persistence
	Internal viscosity	Particle‐tracking microrheology	Increased viscosity is associated with reduced condensate degradability
	Condensate deformability	Optical tweezers or atomic force microscopy (AFM)	Reduced deformability impairs phagophore engulfment
Molecular accessibility	Receptor occupancy	Super‐resolution microscopy or quantitative fluorescence imaging	Higher receptor occupancy promotes cargo recognition and autophagic capture
	Ubiquitin valency	In vitro reconstitution or biochemical assays	Increased ubiquitin multivalency enhances receptor recruitment and condensate organization
Mesoscale organization	RB1CC1 recruitment	Live‐cell fluorescence imaging	Efficient RB1CC1 recruitment promotes autophagosome initiation
	MAP1LC3 recruitment	Fluorescence microscopy or live‐cell imaging	Increased MAP1LC3 recruitment predicts productive phagophore assembly
Membrane coupling	Membrane contact angle (wetting)	Cryo‐electron tomography (Cryo‐ET)	Improved membrane wetting facilitates condensate engulfment
Autophagic outcome	Lysosomal delivery	Tandem fluorescent reporters (e.g., GFP‐mCherry assays)	Efficient lysosomal delivery predicts productive degradation
	Autophagic flux	MAP1LC3‐II turnover and SQSTM1 degradation assays	Higher autophagic flux reflects efficient condensate clearance

**TABLE 2:** Evidence hierarchy and experimental context for representative condensate‐autophagy examples.

Representative example (Ref)	Evidence for condensate formation/LLPS	Evidence for autophagic function	Experimental context	Interpretation level	Caution/wording recommendation
SQSTM1‐polyubiquitin condensates [19]	K63‐linked polyubiquitin promotes SQSTM1 phase separation; evidence includes cell‐based puncta/condensates, valency dependence, dynamic condensate behavior, and in vitro/biochemical support in the study.	Supports autophagic cargo segregation and recruitment of autophagy machinery; productive clearance should be interpreted together with cargo turnover/flux assays rather than MAP1LC3 or SQSTM1 colocalization alone.	Mammalian cells; cell‐based and biochemical/in vitro assays.	Strong evidence for SQSTM1‐polyubiquitin condensate formation; functional autophagy evidence is context‐dependent.	Use “phase‐separated SQSTM1 condensates” where LLPS assays are present; avoid equating SQSTM1 puncta alone with degradation.
SQSTM1 filaments presenting ubiquitinated cargo [20]	Reconstitution/biochemical evidence that SQSTM1 forms filamentous assemblies crosslinked by polyubiquitin; provides mechanistic support for higher‐order cargo organization.	Shows cargo presentation to MAP1LC3‐family proteins and autophagy machinery; primarily supports cargo recognition and presentation rather than proving complete degradation by itself.	In vitro reconstitution/biochemical systems with supporting cellular relevance.	Strong mechanistic evidence for scaffold organization; autophagic degradation requires cellular flux/cargo‐turnover validation.	Best described as reconstituted SQSTM1 filament/condensate organization, not direct endogenous mammalian flux evidence.
NBR1/TAX1BP1‐assisted SQSTM1 ubiquitin condensates [78]	Reconstitution defines how SQSTM1, NBR1, and TAX1BP1 cooperate in ubiquitin condensate formation and initiation‐factor recruitment.	Connects ubiquitin condensates to autophagy initiation, including receptor/scaffold recruitment; provides mechanistic support for initiation rather than requiring inference from colocalization alone.	In vitro reconstitution plus mammalian‐cell relevance.	Strong mechanistic evidence for condensate‐scaffold organization and initiation coupling.	Distinguish reconstituted initiation mechanisms from endogenous mammalian flux unless directly assayed.
TAX1BP1 threshold licensing of aggrephagy [80]	Analyzes SQSTM1/NBR1 cargo condensates and threshold‐dependent TAX1BP1 recruitment rather than establishing LLPS solely from puncta.	TAX1BP1 recruitment advances aggrephagy from cargo collection toward sequestration by promoting assembly of TBK1‐, RB1CC1‐, and ATG9‐positive machinery.	Mammalian‐cell aggrephagy models with mechanistic cell biology.	Strong evidence for receptor‐threshold licensing and sequestration steps.	Use “recruitment/transition toward sequestration” unless degradation/flux endpoints are explicitly measured in the specific experiment.
RB1CC1 initiation assemblies induced by ER‐surface Ca^2+^ transients [65]	Starvation/Torin1/Ca2^+^ perturbation induces dynamic, fusion‐prone RB1CC1 puncta; evidence includes live‐cell multi‐SIM, fusion/coalescence, FRAP recovery, 1,6‐hexanediol sensitivity, Ca^2+^ dependence, and endogenous RB1CC1 puncta in some assays.	RB1CC1 puncta associate with ER and MAP1LC3 structures; EI24/Ca^2+^ perturbations alter RB1CC1/WIPI2/MAP1LC3 puncta, tandem MAP1LC3 reporter readouts, autophagic‐structure maturation, and TEM‐detected autophagic structures.	Mammalian COS7 and other cells; tagged RB1CC1 systems, endogenous markers, EI24 KO, Ca^2+^ pharmacology, TEM.	Strong evidence for liquid‐like RB1CC1 assemblies under defined conditions; endogenous physiological ULK1‐RB1CC1 LLPS should still be stated cautiously.	Do not generalize all endogenous ULK1‐RB1CC1 puncta as physiological LLPS; label as “condensate‐like” where criteria are incomplete.
TBC1D2B autophagy‐initiation condensates [68]	Reported phase separation of TBC1D2B upon autophagy induction; association with MAP1LC3/GABARAP via LIR and with ATG12 machinery.	Supports early phagophore assembly and self‐limiting turnover of TBC1D2B; evidence is strongest for scaffold/initiation function.	Mammalian autophagy‐induction systems; likely tagged/overexpression plus cellular assays.	Moderate‐to‐strong evidence for an initiation scaffold; exact physiological/endogenous LLPS strength depends on assay context.	Avoid presenting as equivalent to endogenous mammalian LLPS unless endogenous and flux data are explicitly shown.
NCOA4‐ferritin condensates in macro‐ and microferritinophagy [38]	NCOA4 homodimerization and binding to FTH1/FTL drive liquid‐like ferritin‐NCOA4 condensates.	Ferritin‐NCOA4 condensates are routed by macroferritinophagy and microferritinophagy to lysosomal/endolysosomal degradation pathways.	Mammalian ferritinophagy cell models with mechanistic imaging/cell biology.	Strong evidence for condensate‐associated ferritin turnover.	Maintain distinction between NCOA4 condensation and downstream iron‐release/ferroptosis consequences.
Iron‐induced NCOA4 condensation and noncanonical ferritin turnover [39]	Iron loading induces NCOA4 condensation and regulates ferritin fate in an iron‐dependent manner.	Prolonged iron loading redirects ferritin through a TAX1BP1‐dependent, ATG7‐independent noncanonical pathway.	Mammalian iron‐homeostasis/ferritinophagy models.	Strong evidence for iron‐regulated NCOA4 condensation and ferritin routing.	Specify ATG7‐independent/noncanonical context; do not merge with canonical macroautophagy without qualification.
OPTN/CALCOCO2 sheet‐like condensates in Parkin‐mediated mitophagy [74]	OPTN and CALCOCO2 form sheet‐like, liquid‐like condensates on ubiquitinated mitochondria; dynamic/fluid behavior is examined in live cells.	Fluidity is functionally linked to ATG9 vesicle recruitment and mitophagy activity; reducing condensate fluidity suppresses mitophagy.	Mammalian PINK1/PRKN mitophagy models; live‐cell imaging and functional perturbation.	Strong mammalian evidence for adaptor condensates during mitophagy.	Present as Parkin‐dependent mitophagy context; do not generalize to all mitophagy modes.
TBK1‐phosphorylated OPTN filaments with polyubiquitin and MAP1LC3 [44]	TBK1‐phosphorylated OPTN forms filamentous condensates with M1‐linked polyubiquitin and co‐condenses with MAP1LC3‐positive membranes; structural/ultrastructural approaches support membrane association.	Supports cargo sequestration and membrane engagement; strongest for structural coupling rather than complete lysosomal degradation.	Biochemical/structural and mammalian‐relevant systems; cryo‐ET/ultrastructure noted in manuscript discussion.	Strong structural/mechanistic evidence for receptor‐condensate membrane coupling.	Co‐condensation with MAP1LC3 should not be equated with productive autophagic flux unless flux/degradation assays are present.
CCT2‐mediated aggrephagy of low‐liquidity/solid aggregates [81]	CCT2 preferentially associates with low‐liquidity FUS condensates and solid aggregates; in vitro recruitment assays show preference for solid aggregates over liquid granules.	CCT2 binds aggregation‐prone proteins independently of polyubiquitin and interacts with ATG8‐family proteins through a noncanonical VLIR motif; promotes lysosome‐dependent degradation of solid aggregates and reduces endogenous polyQ‐HTT in mouse striatum.	Mammalian cells, in vitro aggregate recruitment, yeast conservation for solid Ape1(P22L), and mouse brain/striatum evidence.	Strong evidence for CCT2 as a solid‐aggregate aggrephagy receptor.	The original study notes unresolved mechanisms for clearance/fragmentation of large inclusion bodies; avoid implying all solid aggregates are readily cleared.
Stress‐granule material‐state transitions and autophagic clearance [45]	Stress granules show LLPS‐like assembly and can mature toward less dynamic states; material‐state evidence varies by study and stress condition.	Autophagy contributes to stress‐granule clearance in defined contexts; TRIM21/G3BP1 and SQSTM1/CALCOCO2 pathways modulate clearance.	Yeast and mammalian stress models; context‐dependent autophagy assays.	Moderate‐to‐strong evidence for autophagic clearance of stress granules; LLPS evidence depends on specific experimental criteria.	MAP1LC3/SQSTM1 association with stress granules should be interpreted as recruitment unless flux/cargo turnover is shown.
HSPA8 condensates in antibacterial xenophagy [77]	HSPA8 forms liquid‐like condensates during intracellular bacterial infection and enriches RHOB and BECN1.	Promotes antibacterial autophagic flux by stabilizing local RHOB‐BECN1 signaling.	Mammalian infection/xenophagy model.	Strong functional evidence for condensate‐assisted xenophagy in this model.	Do not generalize to all xenophagy pathways without additional evidence.
SQSTM1 condensates in lysophagy regulated by HSPB1 [56]^,^ [57]	Damaged lysosomes recruit SQSTM1‐positive bodies/condensates; HSPB1 regulates SQSTM1 condensate organization after lysosomal damage.	Functional lysophagic flux and damaged‐lysosome clearance are supported in the cited studies.	Mammalian lysosomal‐damage models.	Strong evidence for receptor‐body organization and lysophagy; LLPS terminology should follow assays used in each study.	Use “SQSTM1‐positive bodies/condensates” where strict LLPS criteria are incomplete.
TFEB nuclear condensates and autophagy‐lysosome transcription [52, 53, 54]	TFEB undergoes phase separation at CLEAR motif‐containing chromatin regions; material properties and surfactant‐like regulators modulate interfacial behavior and transcriptional output.	Regulates transcription of autophagy–lysosome genes and autophagic/lysosomal capacity; it is not direct cargo degradation.	Mammalian nuclear/transcriptional systems; perturbation of TFEB condensate material properties.	Strong evidence for TFEB phase separation and transcriptional regulation of autophagy–lysosome genes.	Describe as indirect regulation of autophagy capacity, not direct condensate cargo clearance.
PolyQ aggregate material state and autophagic accessibility [46]	Distinguishes non‐fibrillar/amorphous vs. fibrillar polyQ aggregate states by imaging/ultrastructure.	Autophagy preferentially engages/degrades non‐fibrillar polyQ aggregates, whereas fibrillar structures are less productively captured.	Mammalian polyQ disease models with ultrastructural analyses.	Strong evidence that aggregate material state influences autophagic accessibility.	This supports state‐dependent accessibility, but does not mean all solidified aggregates are invisible to autophagy receptors.
Membrane wetting/wrapping of phase‐separated compartments [47, 48]	Physical modeling and selected experimental systems link surface tension, contact angle, wetting state, and membrane mechanics to condensate–membrane interactions.	Predicts complete engulfment, partial wrapping, piecemeal degradation, or stalled association; does not itself prove lysosomal degradation without flux/delivery assays.	Theoretical/physical modeling plus selected experimental observations.	Useful working model for condensate–membrane coupling.	Should be presented as a working model, not a universal rule; receptor density, lipid composition, curvature, and lysosomal capacity may modify outcomes.
Yeast Atg1/PAS phase separation [5, 105]	Atg13‐Atg17 multivalent interactions drive phase separation of the Atg1 complex; PAS condensates are dynamic and coalesce on the vacuolar membrane.	Organizes hierarchical recruitment of downstream Atg proteins and supports autophagosome formation in yeast.	Yeast and in vitro/reconstitution systems.	Strong non‐mammalian mechanistic evidence for LLPS‐based initiation‐site organization.	Valuable for conservation and mechanism, but should not be treated as direct endogenous mammalian evidence.
*C. elegans* PGL granules and autophagic degradation [8]	mTOR regulates phase separation/material state of PGL granules in *C. elegans*.	Material state affects autophagic degradation of PGL granules.	*C. elegans* organismal model.	Strong non‐mammalian evidence for material‐state‐dependent autophagy.	Use as conserved‐principle evidence, not as direct mammalian autophagy evidence.
NUFIP1‐mediated ribophagy [82]	No strong evidence in the cited work that ribosomes undergo LLPS as cargo; mechanism is receptor‐mediated cargo capture.	NUFIP1 links ribosomes to MAP1LC3B during starvation‐induced ribophagy.	Mammalian starvation‐induced ribophagy model.	Strong receptor‐function evidence; not evidence for cargo LLPS.	Use as an example of receptor‐mediated spatial organization rather than phase‐separated cargo condensates.

## 2 Biophysical Layer: Condensate Material States as Determinants of Autophagic Accessibility

In mammalian cells, the susceptibility of biomolecular condensates to autophagic degradation is shaped not only by their molecular composition but also by their physical state. Condensates are dynamic assemblies whose material properties, including internal mobility, molecular packing, porosity, and interface behavior with surrounding membranes, directly influence whether autophagic machinery can access and process them [4]. Rather than being passive cargo bodies, these assemblies behave as physically distinct substrates whose degradability is strongly state‐dependent. This view is increasingly supported by studies showing that even condensates formed by the same proteins may display different autophagic outcomes depending on their liquidity and maturation state.

### 2.1 Molecular Principles Shaping Condensate Accessibility

The accessibility of biomolecular condensates to autophagic machinery is defined by the molecular grammar that governs phase separation—namely, the specific sequence features and interaction rules (such as amino acid composition, charge distribution, and multivalent binding motifs) that determine how biomolecules interact and assemble into condensates. In mammalian cells, intrinsically disordered regions (IDRs), low‐complexity domains, and multivalent interaction motifs provide the weak yet highly cooperative contacts required for condensate assembly [12, 13]. IDRs lack a fixed tertiary structure and are typically enriched in glycine as well as polar and charged residues [14, 15]. Within phase‐separating IDRs, aromatic residues such as tyrosine and phenylalanine frequently function as interaction “stickers,” whereas surrounding flexible residues act as “spacers,” together determining the threshold, density, and molecular permeability of the condensed phase [12, 16]. Electrostatic, *π–π*, cation‐*π*, and transient hydrophobic interactions collectively regulate condensate viscosity, porosity, and internal molecular exchange dynamics [12, 17, 18]. Thus, condensate accessibility should be understood not as an intrinsic fixed property, but as a direct molecular consequence of interaction valency, scaffold architecture, and client composition.

The SQSTM1/p62 (sequestosome 1)‐polyubiquitin system provides a paradigmatic example of how interaction grammar defines condensate accessibility (Figure 2). Through PB1‐mediated self‐assembly and multivalent recognition of K63‐linked ubiquitin chains via its UBA domain, SQSTM1 drives the formation of condensates that concentrate ubiquitinated cargo [19]. This mechanism was demonstrated using a combination of mammalian cell‐based imaging, biochemical phase‐separation assays, and purified reconstitution systems, in which K63‐linked polyubiquitin promoted SQSTM1 condensation, whereas monoubiquitin failed to induce comparable assembly [19]. The inability of monoubiquitin to trigger condensation, together with the strong valency dependence observed for longer ubiquitin chains, indicates that multivalent ubiquitin scaffolding establishes the phase‐separation threshold and molecular accessibility of these assemblies [19]. Reconstitution analyses further showed that SQSTM1 phase separation is organized through filamentous SQSTM1 assemblies that are crosslinked by polyubiquitin chains acting as physical bridges between SQSTM1 oligomers [20]. In this minimal biochemical system, efficient cluster formation required cargoes bearing at least two ubiquitin chains longer than three ubiquitin moieties, thereby defining a quantitative threshold for selective cargo capture and mesoscale scaffold assembly [20].

**FIGURE 2:**
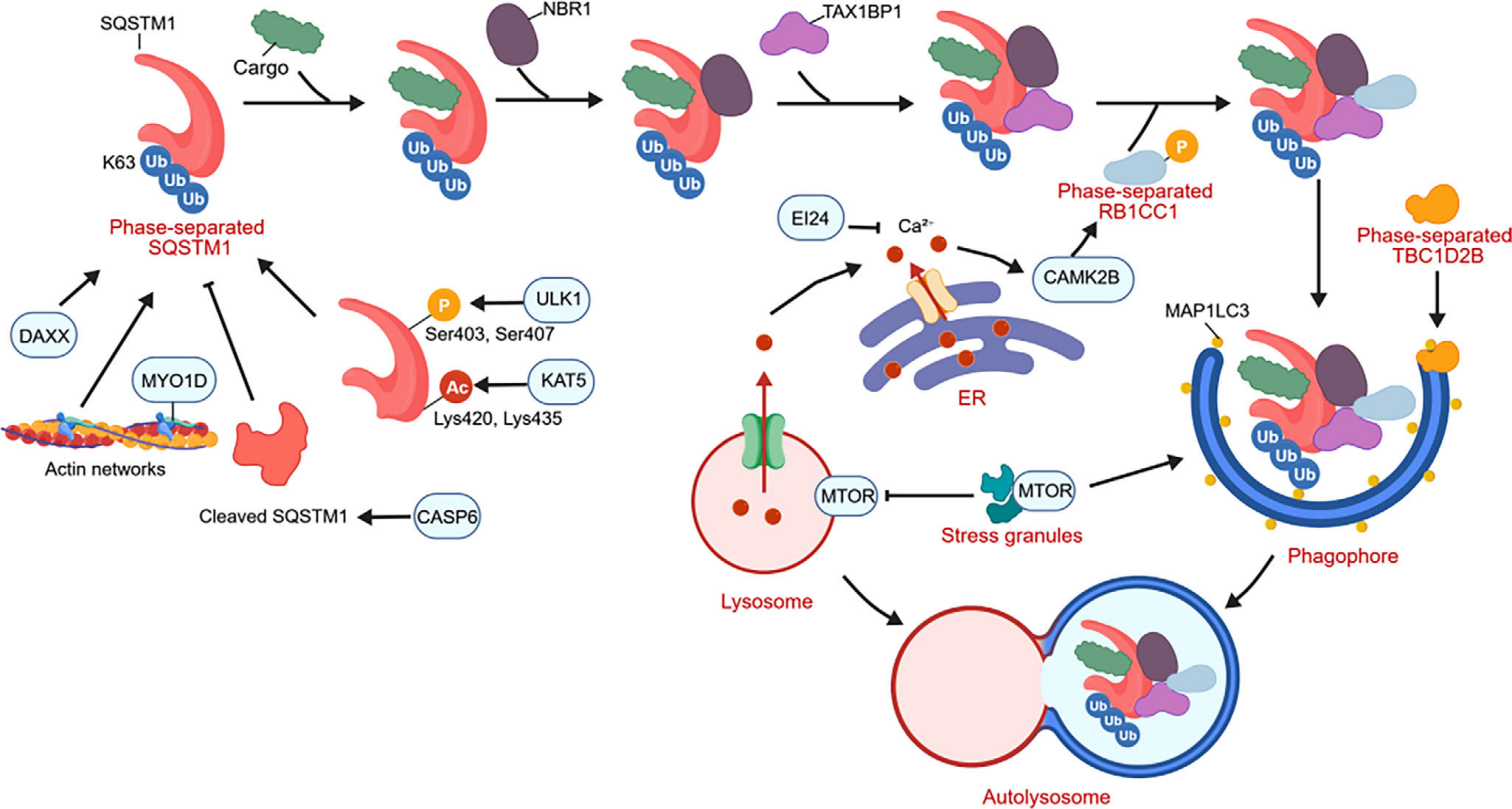
Phase separation‐guided organization of SQSTM1 condensates and autophagy initiation. SQSTM1 undergoes phase separation with K63‐linked polyubiquitin to form condensates that concentrate ubiquitinated cargo. NBR1 enhances condensate assembly and recruits TAX1BP1, linking cargo to RB1CC1‐mediated autophagy initiation. SQSTM1 condensation is regulated by post‐translational modifications, including ULK1‐dependent phosphorylation and KAT5/TIP60‐mediated acetylation, and by cytoskeletal dynamics. Nutrient deprivation and MTORC1 inhibition trigger ER Ca^2^^+^ transients that promote RB1CC1 condensate formation, with CAMK2B acting as a signal decoder and EI24 limiting Ca^2^^+^ release. TBC1D2B forms phase‐separated condensates that facilitate early phagophore assembly through interactions with LC3/GABARAP and ATG12 machinery. Stress granules sequester MTOR, contributing to autophagy activation. Cargo‐containing condensates are ultimately engulfed by the phagophore and degraded upon autolysosome formation.

SQSTM1 condensates do not merely concentrate generic ubiquitinated substrates. The vault complex, a large barrel‐shaped ribonucleoprotein assembly, is identified as a selective cargo recruited into SQSTM1 bodies through direct interaction between major vault protein and NBR1 (NBR1 autophagy cargo receptor), thereby refining cargo specificity and condensate accessibility [21]. This finding supports the notion that condensates function as selective mesoscale sorting compartments rather than passive deposition sites. In this context, K63‐linked ubiquitin functions not simply as a degradation signal, but as an organizational code that integrates cargo recognition, scaffold architecture, and downstream autophagic fate.

A broader mechanistic framework is provided by UBQLN2 (ubiquilin 2), in which ubiquitin acts as a reversible phase switch. Using purified recombinant UBQLN2 together with mono‐ and polyubiquitin chains in in vitro phase‐separation assays, complemented by mammalian cell imaging, UBQLN2 undergoes robust LLPS through weak multivalent self‐interactions under basal conditions [22]. By contrast, mono‐ and polyubiquitin binding disrupt these interactions and reverse LLPS, shifting UBQLN2 from the condensed to the dispersed phase [22]. This reversible transition likely facilitates client extraction and shuttling during protein quality control. Further refinement comes from the observation that distinct polyubiquitin chain topologies exert different effects despite comparable binding affinities: extended K63‐linked and M1‐linked chains promote LLPS, whereas compact K48‐linked and K11‐linked chains inhibit condensation [23]. These findings indicate that ubiquitin linkage architecture itself encodes condensate material properties and accessibility.

Structural and in situ ultrastructural studies further refine this notion by revealing how protein condensates are physically organized at the mesoscale. Cryo‐electron microscopy analysis reveals that full‐length SQSTM1 assembles into a double‐helical PB1‐domain filament scaffold containing a solvent‐accessible major groove, whereas the flexible C‐terminal region (including the LIR and ubiquitin‐binding modules) remains outwardly exposed [24]. This architecture provides a direct structural basis for condensate accessibility, allowing dynamic engagement with both polyubiquitin chains and MAP1LC3/LC3 (microtubule‐associated protein 1 light chain 3)‐family proteins. Polyubiquitin promotes filament bundling and higher‐order condensate assembly, whereas MAP1LC3 binding induces filament shortening and partial disassembly, indicating that condensate scaffold architecture is actively remodeled during cargo processing and phagophore engagement [24].

Beyond scaffold architecture, cytoskeletal dynamics provide an additional layer of spatiotemporal control over condensate maturation. ACTR2/ARP2 (actin‐related protein 2)‐ and ACTR3/ARP3‐dependent branched actin networks and MYO1D (myosin ID) drive the coalescence of nanoscale SQSTM1 puncta into micron‐scale condensates, thereby facilitating sequestration into phagophores that subsequently mature into autophagosomes [25]. Disruption of this branched actin network stalls SQSTM1 bodies in a smaller, poorly degradable state, suggesting that cytoskeletal coupling is a critical determinant of condensate maturation and degradative competence [25].

Protein‐RNA condensates provide another instructive example of how molecular composition governs accessibility. Using mammalian cell stress models, purified protein‐RNA reconstitution systems, and live‐cell imaging, stress granules assembled by proteins such as G3BP1 (G3BP stress granule assembly factor 1) and FUS (FUS RNA binding protein) have been shown to illustrate how RNA concentration, interaction valency, and RNA sequence architecture collectively determine condensate permeability and receptor accessibility [26, 27]. Biochemical reconstitution together with fluorescence recovery after photobleaching (FRAP) analyses further demonstrated that altered RNA composition or disease‐associated mutations increase intermolecular crosslinking, reduce internal molecular exchange, and progressively restrict condensate accessibility [26, 28]. These findings support a broader principle in which client composition encodes condensate accessibility.

Post‐translational modifications introduce an additional regulatory mechanism that actively rewires condensate accessibility. Distinct modifications exert mechanistically divergent effects on condensate state and degradability. Phosphorylation of SQSTM1 at Ser403 and Ser407 enhances polyubiquitin binding and lowers the threshold for phase separation [29, 30], whereas phosphorylation at Ser349 remodels client retention by strengthening KEAP1 (kelch‐like ECH‐associated protein 1) binding and reducing molecular efflux [30]. ULK1 (unc‐51 like autophagy activating kinase 1) localizes within SQSTM1 bodies and directly catalyzes these modifications, indicating that condensate‐resident kinase activity actively reshapes both condensate accessibility and downstream signaling output [30]. KAT5/TIP60 (lysine acetyltransferase 5)‐mediated acetylation of Lys420 and Lys435 within the UBA domain disrupts UBA dimerization and enhances polyubiquitin binding, thereby promoting SQSTM1 phase separation and the formation of SQSTM1 condensates that are subsequently cleared by autophagy [31]. By contrast, proteolytic cleavage by CASP6 (caspase 6) generates a dominant‐negative SQSTM1 fragment that suppresses phase separation and impairs droplet‐based autophagosome formation [32].

Additional regulatory inputs operate at multiple levels of condensate assembly. Evidence from mammalian cell models, purified protein reconstitution, and FRAP analyses indicates that cytoplasmic DAXX (death domain‐associated protein) promotes SQSTM1 oligomerization and condensate nucleation, thereby enhancing KEAP1 recruitment and NFE2L2/NRF2 (NFE2‐like bZIP transcription factor 2) activation [33]. Likewise, genetic ablation of *Nbr1* in mouse embryonic fibroblasts, combined with oxidative stress models and in vitro reconstitution assays, demonstrated that NBR1 stabilizes SQSTM1 liquid condensates, facilitates KEAP1 sequestration, and prolongs antioxidant signaling [34].

Condensate accessibility therefore emerges as a hierarchical property shaped by the integration of interaction grammar, scaffold architecture, cytoskeletal maturation, receptor cooperativity, and dynamic signaling inputs, rather than by molecular composition alone.

### 2.2 The Material‐State Continuum From Fluid Condensates to Solid Aggregates

The degradability of condensates is shaped not only by molecular composition but also by their material state. In mammalian cells, condensates are best understood as occupying a continuous physical spectrum rather than discrete categories, extending from highly fluid liquid‐like droplets through viscoelastic intermediates to rigid solid‐like inclusions. This transition should be viewed not only as a biophysical classification, but as a continuum of progressively declining autophagic compatibility.

At the fluid end of the spectrum are newly formed liquid‐like condensates characterized by rapid internal molecular exchange, low effective viscosity, spherical morphology, and frequent fusion or coalescence events. FRAP consistently reveals rapid signal recovery in these assemblies, indicating high internal mobility and porosity [35]. Newly formed SQSTM1‐positive ubiquitin condensates exhibit rapid fluorescence recovery and dynamic fusion behavior [19]. These dynamic assemblies are generally permissive to receptor penetration and phagophore engagement, thereby facilitating efficient recruitment of MAP1LC3‐family proteins, RB1CC1/FIP200 (RB1 inducible coiled‐coil 1), and other initiation factors at the cargo surface.

This principle is further illustrated during mitophagy. Phase‐separated SQSTM1 condensates exhibit rapid fusion, fission, and fluorescence recovery, consistent with a highly dynamic liquid‐like state [36]. These condensates physically bridge ubiquitinated mitochondria into grape‐like clusters, suggesting that condensate liquidity directly influences organelle compaction and mesoscale topology during mitochondrial quality control [36]. A complementary mechanism has been demonstrated using mitochondrial depolarization models, purified protein interaction assays, and mammalian cell imaging, in which ubiquitinated NR4A1/NUR77 (nuclear receptor subfamily 4 group A member 1) co‐condenses with SQSTM1 on damaged mitochondria [37]. The intrinsically disordered N‐terminal region of NR4A1 enhances both the magnitude and liquidity of SQSTM1 condensates through interaction with the PB1 domain of SQSTM1, thereby promoting mitochondrial sequestration and autophagic clearance [37].

Ferritinophagy provides another well‐defined example of a degradable liquid‐like condensate state (Figure 3). Extracellular ferric iron (Fe^3^^+^) binds to TF (transferrin) and is internalized through TFRC (transferrin receptor)‐mediated endocytosis. Within endosomes, Fe^3^^+^ is reduced to Fe^2^^+^ by STEAP3 metalloreductase, facilitating its release into the cytosol for cellular utilization. Surplus Fe^2^^+^ is sequestered in ferritin after oxidation to Fe^3^^+^. Using purified proteins, in vitro reconstitution, cryo‐electron tomography, correlative light‐electron microscopy, and mammalian cell models, NCOA4 (nuclear receptor coactivator 4) was shown to promote the formation of liquid‐like ferritin condensates through multivalent interactions mediated by NCOA4 homodimerization and binding to FTH1 (ferritin heavy chain 1) and FTL (ferritin light chain) [38]. These ferritin‐NCOA4 condensates are directly engulfed by both phagophores and endosomes, supporting clearance through macroferritinophagy and microferritinophagy, respectively [38]. Live‐cell imaging combined with iron‐loading and iron‐chelation paradigms further demonstrated that NCOA4 condensation dynamically regulates ferritin fate according to intracellular iron availability [39]. During early iron repletion, NCOA4‐mediated condensation promotes ferritin sequestration and storage, whereas prolonged iron loading redirects ferritin to lysosomes through a TAX1BP1 (Tax1 binding protein 1)‐dependent, ATG7‐independent non‐canonical pathway, illustrating that condensate fate is dynamically rewired according to metabolic context [39].

**FIGURE 3:**
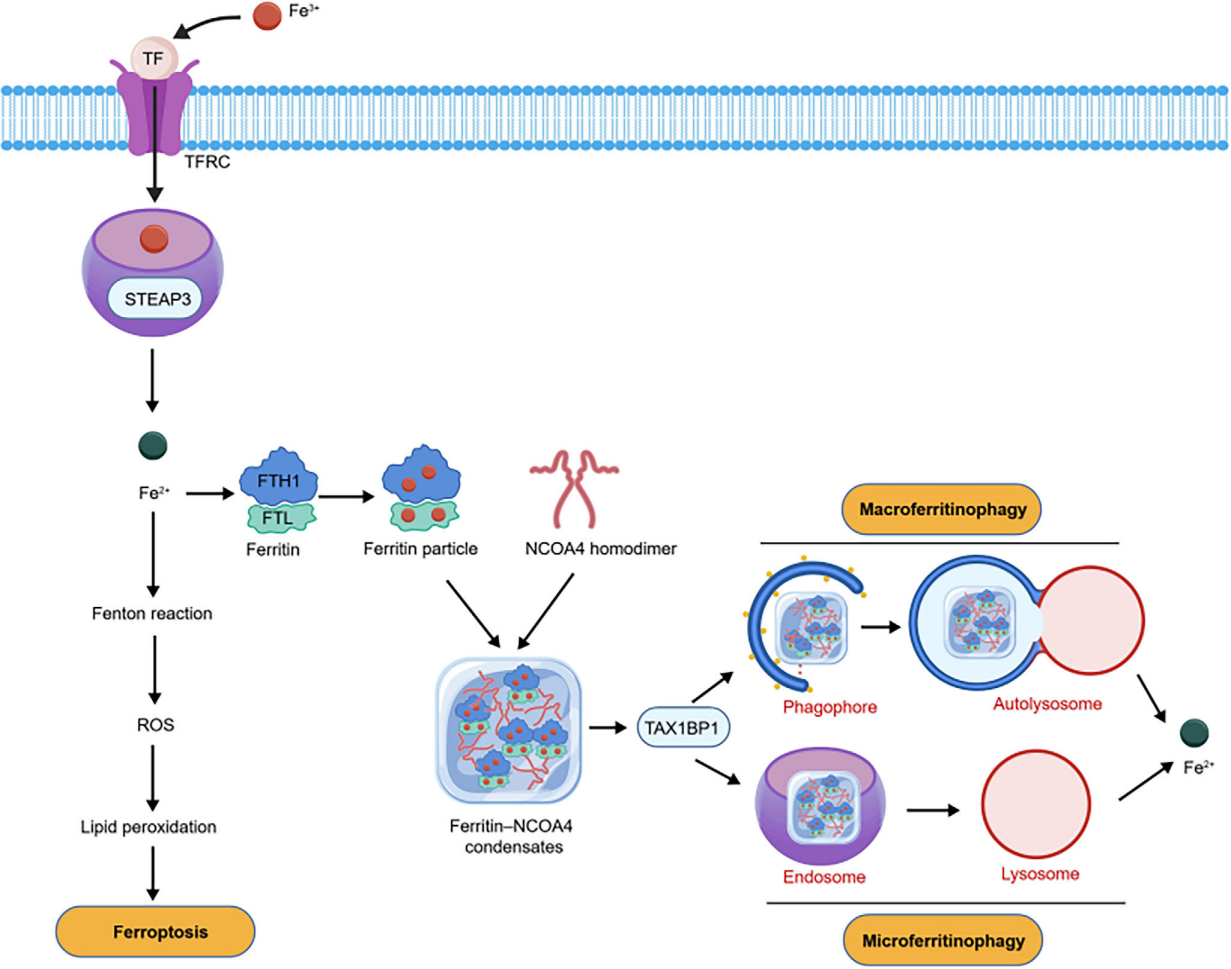
Ferritinophagy, condensate formation, and iron‐dependent ferroptosis. Extracellular ferric iron (Fe^3^^+^) binds TF and is internalized via TFRC‐mediated endocytosis. Within endosomes, Fe^3^^+^ is reduced to Fe^2^^+^ by STEAP3 and released into the cytosol for cellular utilization. Excess Fe^2^^+^ is stored in ferritin following oxidation to Fe^3^^+^. NCOA4 drives the formation of liquid‐like ferritin condensates through multivalent interactions involving NCOA4 homodimerization and binding to FTH1 and FTL. These ferritin‐NCOA4 condensates are targeted for degradation via both macroferritinophagy, in which cargo is engulfed by the phagophore and delivered to autolysosomes, and microferritinophagy through endosomal‐lysosomal pathways. A non‐canonical ferritinophagy pathway that is dependent on TAX1BP1 but independent of ATG7 mediates the turnover of condensate‐associated ferritin. Lysosomal ferritin degradation releases redox‐active Fe^2^^+^, which promotes ROS production through Fenton chemistry, leading to lipid peroxidation and ferroptosis.

Ferritinophagy is increasingly recognized as a key regulatory mechanism in lipid peroxidation‐driven ferroptosis by facilitating lysosomal ferritin degradation, thereby increasing intracellular iron availability and promoting ROS production [40, 41]. Evidence from mammalian ferroptosis models, genetically engineered mice, and disease‐associated tissue models further indicates that NCOA4‐dependent ferritin condensates actively couple iron metabolism with ferroptotic susceptibility [40, 41, 42, 43]. In addition to biochemical regulation, extracellular mechanical cues can further remodel NCOA4‐dependent ferritin condensate organization and ferritinophagic flux, linking matrix stiffness and biomechanical stress to iron homeostasis and ferroptotic sensitivity [42, 43]. Thus, fluid condensates are not only dynamic, but are also physically and functionally compatible with efficient autophagic routing.

Between the fluid and solid extremes lies a broad intermediate regime in which condensates remain only partially degradable. In this viscoelastic or gel‐like state, internal molecular exchange slows but is not abolished, and fusion dynamics become less frequent or incomplete. Phosphorylated OPTN (optineurin) condensates illustrate an internally heterogeneous material state in which the receptor scaffold itself shows minimal fluorescence recovery, whereas co‐condensed polyubiquitin chains retain partial mobility [44]. This mixed physical behavior supports the view that condensates occupy a continuum of states rather than sharply separated categories.

Stress granules under prolonged oxidative or proteotoxic stress provide a representative example of this intermediate regime. Initially reversible and highly dynamic, they progressively acquire increased viscosity and reduced internal mobility, reflecting enhanced intermolecular crosslinking among RNA‐binding proteins and associated RNAs [26, 27, 28]. Regulatory mechanisms continue to operate within this intermediate state. Experimental studies in yeast and mammalian stress models, together with context‐dependent autophagy assays, demonstrated that TRIM21 (tripartite motif containing 21)‐mediated ubiquitination of G3BP1 restrains excessive phase separation, whereas SQSTM1 and CALCOCO2/NDP52 (calcium binding and coiled‐coil domain 2) promote selective autophagic clearance [45]. This state is therefore best interpreted as a delayed yet potentially recoverable degradative state rather than a fully resistant endpoint.

At the opposite end of the continuum lie solid‐like or fibrillar assemblies with minimal internal mobility and markedly reduced molecular permeability. Condensates formed by FUS, TARDBP/TDP‐43 (TAR DNA‐binding protein), and MAPT/tau in neuronal models exemplify this transition. Disease‐associated mutations accelerate the conversion of dynamic droplets into rigid inclusions enriched in β‐sheet interactions and fibrillar architectures [26, 28]. FRAP recovery becomes negligible, molecular penetration is severely restricted, and compatibility with membrane engulfment is substantially reduced. At this stage, the material transition begins to directly bias degradative fate.

Ultrastructural evidence strongly supports this degradability continuum. In mammalian polyglutamine disease models, phagophores engulf the amorphous, non‐fibrillar phase of polyQ aggregates, whereas radially organized amyloid‐like fibrils are largely excluded from productive capture [46]. SQSTM1 selectively associates with the non‐fibrillar aggregate surface, while rigid fibrillar structures exclude receptor binding and frequently trap incomplete autophagic membranes at their periphery [46]. These observations provide structural support for the concept that deformability and material state are major determinants of autophagic accessibility.

Physical models provide one mechanistic framework for interpreting this transition by proposing that condensate‐membrane wetting contributes to condensate degradability (Table 3). Rather than representing a universal mechanism, the membrane‐wetting model should currently be regarded as a working model whose contribution is likely to depend on multiple biophysical and cellular factors. Loss of degradability reflects not only reduced receptor accessibility, but also altered condensate‐membrane wetting behavior and decreased condensate deformability [11]. At the condensate‐phagophore interface, condensate surface tension, membrane interfacial energy, membrane curvature, receptor density, MAP1LC3‐LC3‐interacting region (LIR) interactions, local lipid composition, condensate deformability, and lysosomal competence are proposed to collectively influence the mode and efficiency of cargo sequestration [47]. Within this working model, the effective wetting state (often described by contact angle and interfacial tension) is expected to bias condensates toward complete engulfment, partial wrapping, piecemeal autophagy, or stalled phagophore association rather than determining a single outcome. Low‐surface‐tension droplets, particularly in combination with favorable membrane mechanics, may preferentially support piecemeal autophagy (“fluidophagy”), whereas higher effective surface tension together with appropriate membrane mechanics may favor more complete engulfment [47].

**TABLE 3:** Wetting states and autophagic outcomes.

Dimension	High contact angle (low wetting)	Intermediate contact angle	Low contact angle (high wetting)
Wetting state	Dewetting‐like	Partial wetting	Strong wetting
Condensate properties	Rigid, less deformable, low accessibility	Viscoelastic, partially accessible	Dynamic, deformable, high accessibility
Membrane engagement	Limited contact	Partial wrapping	Extensive wrapping
Sequestration behavior	Inefficient capture; possible escape	Partial enclosure; delayed sequestration	Efficient enclosure; context‐dependent piecemeal or full engulfment
Autophagic outcome (tendency)	Escape/persistence	Delayed degradation	Efficient clearance
Interpretation in this framework	Low membrane compatibility	Intermediate compatibility	High membrane compatibility

In addition, membrane spontaneous curvature and MAP1LC3‐LIR interactions may further influence bending directionality, thereby affecting whether condensates are degraded or instead remain associated with expanding phagophores. Complementary wetting‐based models further propose that condensate autophagy can be described by transitions between dewetting, partial wetting, and complete wetting states, providing a useful conceptual framework for interpreting progressive phagophore enclosure and cargo sequestration [48]. Because the current membrane‐wetting model integrates evidence from physical modeling, in vitro reconstitution, and selected mammalian experimental systems, the corresponding evidence hierarchy and experimental context are summarized in Table 2.

A related heterotypic example is provided by UBQLN2 condensates in Parkinsonian proteinopathy, where SNCA/α‐synuclein fibrillization is accelerated within maturing UBQLN2 droplets [49]. During the liquid‐to‐gel and solid transition of UBQLN2 condensates, SNCA progressively converts into pathogenic fibrils, suggesting that hardening condensates may function not only as poorly degradable endpoints but also as catalytic microenvironments that actively promote client protein fibrillization [49].

This body of evidence supports a state‐dependent clearance model in which fluid condensates are efficiently captured, viscoelastic assemblies exhibit delayed yet potentially recoverable turnover, and rigid inclusions increasingly escape autophagic degradation. Importantly, condensate material state is an important, but not sufficient, determinant of autophagic fate. Instead, condensate degradability emerges from the combined effects of material properties, molecular accessibility, receptor engagement, membrane compatibility, and lysosomal competence. Condensate material state and membrane wetting should therefore be regarded as closely interconnected components of a working model rather than independent or universally applicable determinants of autophagic clearance. Progressive changes from liquid‐like to gel‐like and solid‐like states are expected to alter condensate deformability, molecular accessibility, and membrane compatibility, thereby biasing (rather than solely determining) phagophore engagement and engulfment efficiency.

### 2.3 Aging, Maturation, and Stress‐Driven Hardening

A defining feature of biomolecular condensates is that their material state is not fixed, but dynamically evolves over time and in response to the cellular environment. Accordingly, autophagic accessibility is best understood not as a static property, but as a temporally regulated window that progressively narrows during condensate aging and maturation.

Stress granules provide one of the clearest examples of this time‐dependent transition. Following acute arsenite, heat, or proteotoxic stress, they rapidly assemble into highly dynamic liquid‐like condensates enriched in RNA‐binding proteins, such as G3BP1, FUS, and TARDBP [26, 27, 28]. Under transient stress conditions, these assemblies remain reversible and can be cleared upon stress resolution, in part through selective autophagy pathways. By contrast, prolonged stress exposure, adenosine triphosphate (ATP) depletion, or chronic proteotoxic burden progressively drives their maturation into less mobile and more persistent assemblies. Time‐resolved imaging and FRAP analyses consistently reveal a gradual decline in internal molecular exchange, accompanied by increased intermolecular crosslinking and scaffold entanglement.

Aging further amplifies this process by progressively compromising proteostasis capacity. Declining chaperone activity, reduced ATP availability, impaired proteasomal function, and cumulative oxidative damage collectively promote condensate hardening and reduce reversibility. In neuronal systems, this age‐dependent loss of fluidity is particularly evident in FUS, TARDBP, and MAPT/tau assemblies, which gradually transition from metastable condensates into persistent inclusions enriched in fibrillar architectures and β‐sheet interactions [26, 28]. These persistent assemblies increasingly resist both molecular penetration and membrane engulfment, thereby markedly reducing autophagic turnover.

This maturation process is best viewed not as a passive consequence of elapsed time, but as an active systems‐level response to sustained stress history and declining quality‐control capacity. Chronic oxidative stress, defective chaperone buffering, and recurrent stress‐granule cycling accelerate scaffold hardening and client entrapment, shifting condensates from adaptive transient compartments toward pathological persistence [50].

This active remodeling is further shaped by upstream signaling pathways through post‐translational modification and chaperone‐mediated buffering. Experimental studies in mammalian nuclear transcriptional systems demonstrated that TFEB (transcription factor EB) acts as a key phase‐separating transcriptional regulator of the autophagy‐lysosome pathway. Upon activation, nuclear TFEB undergoes LLPS to form transcriptionally active condensates at CLEAR motif‐containing chromatin regions, thereby promoting the expression of autophagic and lysosomal genes [51, 52, 53, 54]. The material properties of TFEB condensates, including interfacial tension, molecular exchange, and DNA‐binding accessibility, are dynamically regulated by signaling‐dependent inputs such as post‐translational modification and molecular surfactants, directly coupling stress signaling to autophagic output [52, 53, 54]. In parallel, phosphorylation‐dependent remodeling of scaffold proteins, condensate‐associated chaperones, and other regulatory factors, including HNRNPH1 (heterogeneous nuclear ribonucleoprotein H1) and HSPB1/HSP27 (heat shock protein family B (small) member 1), further reshapes condensate dynamics across both cytoplasmic and nuclear autophagy pathways, as demonstrated in mammalian lysosomal‐damage models [55, 56, 57]. Condensate state is therefore not solely determined by intrinsic scaffold properties, but is actively reprogrammed by signaling‐dependent molecular inputs.

In parallel with material hardening, sustained stress also remodels condensate client composition. SQSTM1‐positive assemblies selectively sequester stalled translation‐initiation factors, including EIF2S1/eIF2α (eukaryotic translation initiation factor 2 subunit alpha) and EIF4E/eIF4E (eukaryotic translation initiation factor 4E), together with ribosomal components under conditions of translational arrest and MTOR (mechanistic target of rapamycin kinase) complex 1 (MTORC1) inhibition [58]. Functional analyses indicate that these client proteins undergo SQSTM1‐dependent autophagic degradation, suggesting that prolonged stress reshapes not only condensate material state but also client identity and downstream degradative routing [58]. A conceptually related mechanism is observed in STAU1 (staufen double‐stranded RNA binding protein 1) condensates, which selectively recruit target RNAs and translation machinery, thereby promoting chronic MTOR hyperactivation and downstream autophagic dysfunction in cultured mammalian cells, Huntington's disease knock‐in striatal cells, and patient‐derived fibroblasts [59]. Thus, temporal maturation and client reprogramming are closely coupled processes.

Sustained stress can also drive functional rewiring of condensates. Experimental studies in mammalian inflammatory stress models demonstrated that, under proteotoxic, oxidative, or endotoxin stress, phase‐separated SQSTM1 droplets transition into enlarged SQSTM1‐dependent P‐body‐like assemblies through interaction with the RNA helicase DDX6 (DEAD‐box helicase 6) [60]. In this state, condensates no longer function primarily as degradative scaffolds but instead recruit the inflammasome adaptor PYCARD/ASC (PYD and CARD domain‐containing) to promote NLRP3 (NLR family pyrin domain‐containing 3) inflammasome assembly and inflammation‐associated cytotoxicity [60]. This transition suggests that persistent condensates may undergo identity switching, transforming from adaptive degradative compartments into active inflammatory signaling hubs. A similar principle applies to persistent SQSTM1‐KEAP1 condensates, which can transition from transient degradative assemblies into chronic stress‐signaling hubs when autophagic turnover is impaired [61, 62].

A related example is observed in age‐related macular degeneration, where mitochondrial injury drives redox‐sensitive phase separation of APOE (apolipoprotein E) and SQSTM1, leading to persistent condensate formation in retinal pigment epithelial cells [63]. These condensates correlate with autophagic defects and may contribute to drusen nucleation, further linking mitochondrial stress, defective autophagy, and disease‐associated phase transitions [63].

This places temporal maturation at the center of condensate fate determination: aging progressively converts degradable adaptive assemblies into increasingly persistent and functionally rewired substrates, thereby linking cellular stress history, declining proteostasis capacity, and disease progression.

## 3 Organizational Layer: Condensates as Mesoscale Reaction Fields for Autophagy

Beyond determining degradability, phase separation also provides a higher‐order organizational principle for autophagy. In mammalian cells, condensates create spatially restricted reaction fields that locally concentrate signaling factors, receptors, membrane sources, and cargo interfaces, thereby coordinating the stepwise assembly of autophagic machinery. This mesoscale organization is particularly important for selective autophagy, where productive degradation depends on precise spatiotemporal coupling between cargo recognition and phagophore formation.

### 3.1 Condensate‐Assisted Organization of Autophagy Initiation

Autophagy initiation in mammalian cells is increasingly understood as a spatially organized process involving condensate‐like assembly of the ULK1‐ATG13 (autophagy‐related 13)‐RB1CC1 initiation machinery. Recent studies demonstrate higher‐order assembly of RB1CC1 and, under defined experimental conditions [64], support the formation of liquid‐like RB1CC1 condensates that promote local concentration of autophagy factors and phagophore nucleation. However, the extent to which endogenous ULK1‐RB1CC1 assemblies fulfill the stringent criteria for physiological LLPS remains to be fully established. Accordingly, throughout this review we distinguish condensate‐like assemblies from experimentally validated LLPS where appropriate and emphasize the functional consequences of mesoscale organization rather than a strict classification of assembly states. The conceptual framework proposed here does not require every representative initiation assembly to satisfy all experimental criteria for LLPS. Rather, it emphasizes the common organizational principle that higher‐order molecular assemblies spatially coordinate cargo recognition, autophagy initiation, and membrane remodeling, irrespective of whether they are experimentally validated LLPS or condensate‐like assemblies. The strength and experimental context of the available evidence supporting representative initiation condensates are summarized in Table 2.

A central regulatory input is localized Ca^2^^+^ signaling at the ER surface [65], to which lysosomal Ca^2^^+^ release represents one contributing source [66] (Figure 2). ER Ca^2^^+^ transients can be triggered by nutrient deprivation and MTORC1 inhibition, and are mediated by ER Ca^2^^+^ release channels. Experimental studies in mammalian COS7 and other cell models, using tagged RB1CC1 systems, endogenous markers, Ca^2^^+^ pharmacological perturbation, and transmission electron microscopy (TEM), demonstrated that these transient Ca^2^^+^ elevations promote the nucleation, fusion, and coalescence of small RB1CC1‐positive assemblies, which grow until reaching a size threshold compatible with autophagosome initiation [65]. Live‐cell super‐resolution imaging shows that these RB1CC1 puncta are highly fusion‐prone and dynamically coalesce along ER tubules, supporting a liquid‐like phase‐separation mechanism [65]. Once established, these initiation condensates concentrate ULK1 complex components, facilitate kinase activation, and promote recruitment of downstream factors such as the PIK3C3/VPS34 (phosphatidylinositol 3‐kinase catalytic subunit type 3)‐containing complex and phosphatidylinositol‐3‐phosphate/PtdIns3P‐enriched membranes [65].

This mechanism establishes a link between local biochemical signaling and membrane biogenesis. By confining autophagy initiation to condensate‐defined ER subdomains, cells ensure that phagophore formation occurs only when sufficient molecular density and signaling inputs are locally achieved. In this sense, RB1CC1 condensates function as spatial thresholds that convert transient biochemical signals into stable autophagosome initiation sites.

This framework is further reinforced by upstream signal decoding mechanisms (Figure 2). CAMK2B/CaMKIIβ (calcium/calmodulin dependent protein kinase II beta) acts as a molecular decoder of starvation‐induced ER Ca^2^^+^ transients by directly phosphorylating RB1CC1 at Thr269, Thr1127, and Ser1484 and modulating its phase‐separation behavior [67]. Loss of CAMK2B reduces RB1CC1 puncta formation and disrupts their progression into functional initiation sites, indicating that condensate assembly is actively regulated by local signaling dynamics [67]. In contrast, EI24 (EI24 autophagy‐associated transmembrane protein) suppresses excessive ER Ca^2^^+^ transients, thereby preventing the aberrant accumulation of RB1CC1 condensates at the ER [66].

A parallel scaffold‐centered mechanism operates at the early stage of autophagosome biogenesis. TBC1D2B (TBC1 domain family member 2B) undergoes phase separation into liquid‐like droplets upon autophagy induction and directly interacts with MAP1LC3 and GABARAP (GABA type A receptor‐associated protein) proteins through a canonical LIR motif while associating with the ATG12 conjugation machinery [68]. These condensates function as localized initiation platforms for early phagophore assembly. The subsequent autophagic degradation of TBC1D2B itself further suggests a self‐limiting initiation mechanism that couples early scaffold formation with downstream turnover.

A complementary mechanism operates at cargo surfaces during selective autophagy. Multivalent low‐affinity interactions between autophagy receptors and cargo surfaces promote receptor mobility, which is required for formation of discrete initiation hubs [69]. Rather than uniformly coating cargo, receptors and early initiation factors assemble into dynamic condensate‐like foci that subsequently coalesce into mature phagophore assembly sites [69]. This establishes a key principle: selective autophagy initiation depends not simply on receptor binding strength, but on a dynamically organized reaction field generated by reversible multivalent interactions at the cargo interface.

Spatial organization of initiation is further shaped by organelle architecture. During early starvation responses, RAB5A/RAB5 (RAB5A, member RAS oncogene family)‐ and RAB11A/RAB11‐positive endosomes are dynamically tethered to ER exit sites, creating a confined microenvironment that promotes local Ca^2^^+^ accumulation and phase transition of SEC16A (SEC16 homolog A, endoplasmic reticulum export factor)‐positive components [70]. This spatially restricted reaction field facilitates condensate formation and generation of membrane material that seeds de novo phagophore assembly [70]. These observations suggest that condensate formation is instructed not only by molecular concentration thresholds but also by membrane contact architecture.

In addition to serving as localized initiation platforms, phase‐separated condensates can also regulate autophagy at the signaling level. For example, condensates such as stress granules are capable of sequestering MTOR, thereby promoting its dissociation from the lysosomal surface, suppressing MTORC1 activity, and consequently activating autophagy (Figure 2) [71, 72]. Together, these findings highlight that condensates orchestrate autophagy initiation through multiple mechanisms, including the spatial organization of initiation factors, the coordination of membrane remodeling, and the modulation of upstream signaling pathways.

### 3.2 Organelle‐ and Cargo‐Centered Reaction Fields

A related principle is observed in ERphagy/reticulophagy, where localized Ca^2^^+^ microdomains generated by ER‐resident mechanosensory channels promote robust RB1CC1 phase separation at ER subdomains [73]. These condensates function as spatially restricted initiation platforms that couple local stress sensing to selective sequestration of ER fragments [73]. This mechanism highlights how condensate formation can coordinate both initiation‐site specification and membrane‐source selection.

A conceptually related but receptor‐centered mechanism operates in PINK1 (PTEN‐induced kinase 1) and PRKN/PARKIN (parkin RBR E3 ubiquitin protein ligase)‐dependent mitophagy. OPTN and CALCOCO2 assemble into sheet‐like liquid condensates on the surface of ubiquitinated damaged mitochondria rather than forming stoichiometric receptor coats [74]. Live‐cell imaging and FRAP analyses reveal rapid molecular exchange and dynamic redistribution of these receptor condensates, particularly at mitochondria‐phagophore contact sites [74]. Reducing condensate fluidity suppresses ATG9 vesicle recruitment, impairs RB1CC1 accumulation, and blocks mitophagy initiation, indicating that receptor liquidity is functionally required for reaction‐field formation [74].

This condensate behavior is further reinforced by receptor‐intrinsic regulation. OPTN itself undergoes phosphorylation‐dependent LLPS, driven by Ser173 phosphorylation within its intrinsically disordered N‐terminal region, which stabilizes its association with phagophore membranes and promotes co‐condensation with ubiquitin‐modified mitochondria through its C‐terminal ubiquitin‐binding domain [75]. In this way, phosphorylation functions as a molecular switch that enhances the scaffold capacity of receptor condensates during mitophagy initiation.

A complementary damage‐sensing pathway operates upstream of receptor condensate assembly. Following rupture of the mitochondrial outer membrane, LGALS3 (galectin 3) forms condensate‐like assemblies on injured mitochondria that facilitate ULK1 recruitment and local initiation‐site formation [76]. In this setting, condensate formation functions as a damage‐sensing platform that converts membrane rupture signals into spatially restricted autophagy initiation.

A parallel condensate‐assisted mechanism also operates in xenophagy. During intracellular bacterial infection, HSPA8 (heat shock protein family A (Hsp70) member 8) forms liquid‐like condensates that concentrate RHOB (ras homolog family member B) and BECN1 (beclin 1) within phase‐separated droplets [77]. This local enrichment stabilizes RHOB‐BECN1 interactions and promotes antibacterial autophagic flux [77], extending the mesoscale reaction‐field principle to host‐defense autophagy.

An unresolved question is whether common physical rules govern the spatial scale, boundary stability, and membrane selectivity of these organelle‐ and cargo‐centered reaction fields across distinct forms of selective autophagy. How local lipid composition, membrane curvature, and receptor valency collectively define the transition from condensate assembly to productive phagophore engagement remains incompletely understood.

### 3.3 Selective Autophagy Receptors as Scaffold‐Adapter Systems

At the level of cargo recognition, selective autophagy receptors function not merely as molecular adaptors but as organizational modules that convert substrate recognition into localized autophagosome biogenesis. In mammalian cells, receptors such as SQSTM1, NBR1, OPTN, TAX1BP1, and CCT2 (chaperonin‐containing TCP1 subunit 2) collectively establish a scaffold‐receptor system that couples cargo clustering to phagophore initiation and membrane engagement [78].

The SQSTM1‐centered ubiquitin condensate system provides one of the clearest examples of mesoscale reaction‐field organization, in which SQSTM1‐ubiquitin assemblies locally concentrate cargo, receptors, and early initiation factors [19, 20, 79]. These condensates function as dynamic scaffolds rather than passive storage sites. A central step in this process is the direct interaction between SQSTM1 and the claw domain of RB1CC1, which links cargo‐associated condensates to the ULK1 initiation complex and promotes local phagophore formation [64]. Reconstitution analyses further show that this interaction is selective for cargo‐associated SQSTM1 assemblies, thereby restricting RB1CC1 recruitment to ubiquitin‐rich condensates rather than allowing diffuse cytosolic engagement [64]. This provides a mechanistic explanation for how condensate formation licenses spatially confined phagophore nucleation.

Within this framework, receptor cooperation introduces an additional model of mesoscale organization. NBR1 cooperates with SQSTM1 through PB1 domain‐mediated association and enhances condensate formation by contributing a high‐affinity UBA domain, thereby increasing the efficiency of ubiquitin cargo clustering [78]. In parallel, NBR1 functions as a bridging factor that recruits TAX1BP1 into SQSTM1‐ubiquitin condensates [78, 80]. TAX1BP1 in turn acts as a connector to RB1CC1, functionally coupling cargo condensation to upstream autophagy initiation [78]. This division of labor defines a hierarchical scaffold‐receptor system in which SQSTM1 primarily drives condensate assembly, NBR1 stabilizes and amplifies cargo clustering, and TAX1BP1 strengthens the connection to initiation machinery.

This organizational logic extends beyond aggrephagy. A parallel principle is evident in OPTN‐dependent selective autophagy, particularly during mitophagy. TBK1 (TANK binding kinase 1)‐phosphorylated OPTN forms filamentous condensates with M1‐linked polyubiquitin that directly co‐condense with MAP1LC3‐positive membrane structures [44]. Cryo‐electron tomography further shows that these assemblies physically align and deform MAP1LC3‐coated vesicles, providing structural evidence that receptor condensates spatially coordinate cargo sequestration with nascent phagophore membrane engagement [44]. This suggests that receptor‐driven condensates not only cluster substrates but also actively organize membrane topology during selective cargo capture.

Receptor specialization further refines this notion at later stages of condensate maturation. Experimental studies in mammalian cells, supported by in vitro aggregate recruitment assays, yeast conservation analyses, and mouse brain/striatum models, identified CCT2 as an aggrephagy receptor specialized for condensates with reduced liquidity and solid protein aggregates [81]. Unlike canonical ubiquitin‐binding receptors such as SQSTM1, NBR1, and TAX1BP1, which target more fluid or partially dynamic condensates, CCT2 binds aggregation‐prone proteins independently of ubiquitination and engages MAP1LC3‐family proteins through a non‐canonical VLIR motif [81]. This specialization extends condensate surveillance across the material‐state continuum and highlights that distinct receptors may preferentially recognize condensates with different physical properties. A schematic overview of receptor preference and degradability across the liquid–gel–solid condensate continuum is presented in Figure 4.

**FIGURE 4:**
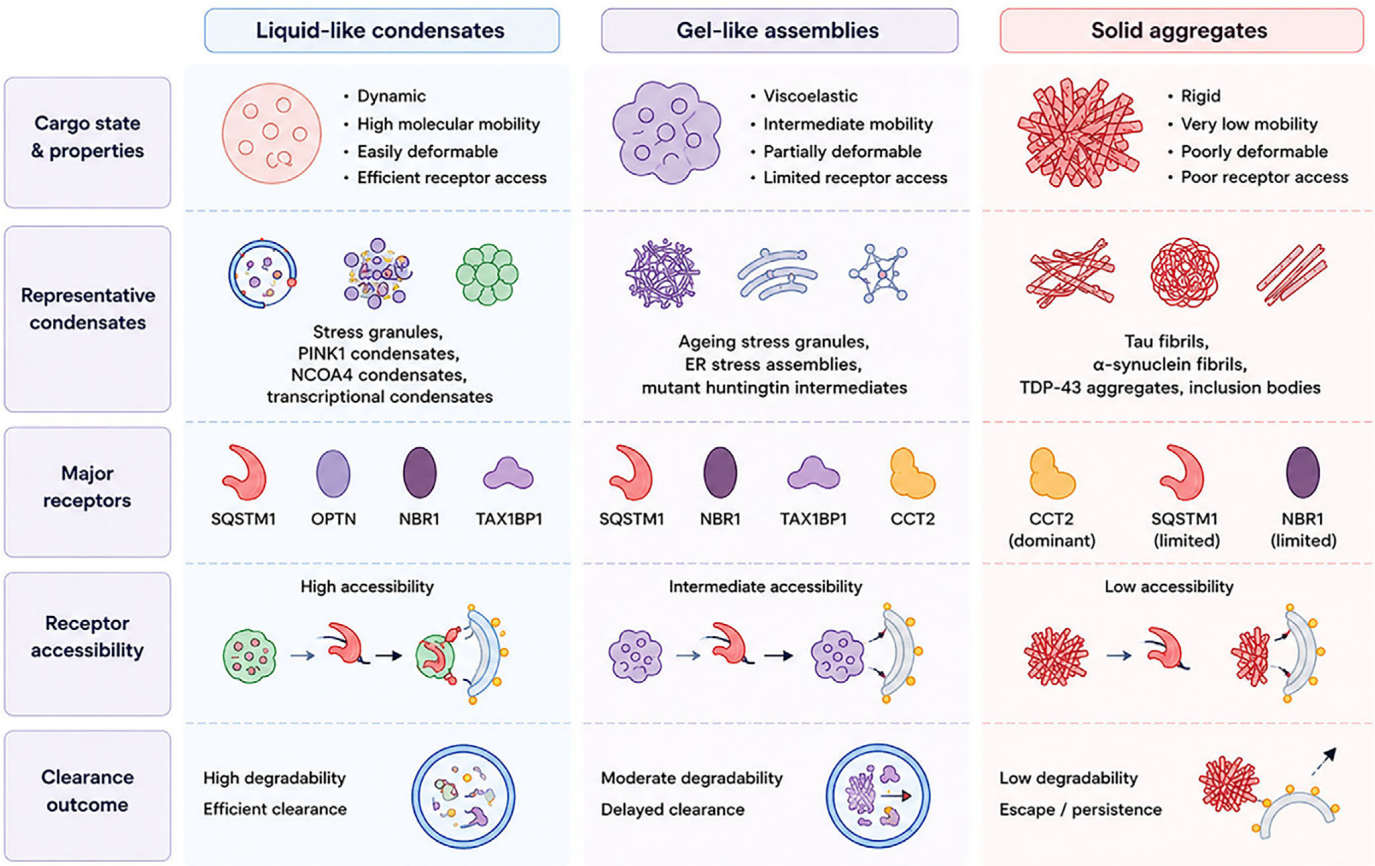
Aggrephagy across the condensate material‐state continuum. Biomolecular cargos exist along a continuum from liquid‐like condensates to gel‐like assemblies and solid aggregates. Progressive condensate maturation is accompanied by reduced molecular mobility, deformability, and receptor accessibility, resulting in a gradual decline in degradability. Different selective autophagy receptors preferentially recognize cargos with distinct material properties. Dynamic condensates are recognized by receptors including SQSTM1, OPTN, NBR1, and TAX1BP1, whereas progressively hardened assemblies increasingly require specialized receptors such as CCT2. These state‐dependent differences ultimately determine phagophore engagement, cargo sequestration, and autophagic clearance, leading to efficient degradation, delayed turnover, or persistence of poorly degradable aggregates.

Although aggrephagy and mitophagy currently provide the strongest mechanistic evidence linking condensate organization to selective autophagy, emerging studies suggest that similar organizational principles extend to ferritinophagy and additional selective autophagy pathways [38]. During xenophagy, condensate‐assisted enrichment of pathogen‐sensing and autophagy regulators facilitates localized cargo recognition and autophagosome assembly, as illustrated by HSPA8 liquid‐like condensates that concentrate RHOB and BECN1 to promote antibacterial autophagic flux [77]. In lysophagy, damaged lysosomes recruit SQSTM1‐positive bodies and associated receptor assemblies, whose oligomerization and HSPB1‐regulated organization facilitate lysophagic flux and damaged‐lysosome clearance [57]. By contrast, ERphagy and ribophagy appear to rely less on established cargo phase separation and more on receptor‐mediated spatial organization. ERphagy employs ER‐resident receptors to coordinate selective membrane remodeling and sequestration [65], whereas NUFIP1 (nuclear FMR1 interacting protein 1) links ribosomes to MAP1LC3B during starvation‐induced ribophagy [82]. Collectively, these findings suggest that, despite differences in cargo identity and molecular machinery, receptor‐mediated mesoscale organization represents a common organizational strategy underlying diverse forms of mammalian selective autophagy.

These principles are broadly consistent with observations in yeast, *C. elegans*, and plants, where multivalent scaffold assembly likewise couples cargo recognition to autophagosome formation [5, 6, 7, 8, 9]. Mammalian systems, however, incorporate additional regulatory layers, including post‐translational modifications, stress signaling, and expanded receptor networks, that confer greater plasticity and context dependence on condensate‐mediated selective autophagy.

Together, these findings support a unified view in which selective autophagy receptors function as an integrated scaffold‐receptor network that translates molecular recognition into mesoscale organization, thereby coupling cargo selection with spatially restricted autophagosome biogenesis and membrane engagement across diverse selective autophagy pathways. Representative experimental evidence supporting these receptor‐condensate systems, together with their corresponding experimental contexts, is summarized in Table 2.

### 3.4 Spatial Reaction Fields for Cargo Processing and Machinery Coordination

Once formed, condensates function as spatial reaction centers in which multiple steps of the autophagy pathway are coordinated. Within these assemblies, cargo molecules are concentrated, receptors are oligomerized, and initiation complexes are recruited in close proximity. This spatial confinement increases the effective concentration of interacting components and stabilizes otherwise transient interactions through multivalent binding. More broadly, membrane surfaces act as two‐dimensional biochemical scaffolds that further enhance local reactant concentration, reduce the entropic cost of molecular encounters, and spatially align interaction partners [83]. During autophagosome biogenesis, receptor condensates and the growing phagophore membrane together establish a coupled reaction field that promotes avidity‐driven assembly of cargo‐engulfment and initiation machinery [83].

A central physical principle underlying this organization is avidity. Phase separation converts multiple individually weak interactions into a collectively stable and persistent binding platform. Within cargo‐associated condensates, clustered ubiquitin chains, oligomerized receptors, and scaffold proteins are brought into close proximity, thereby stabilizing interactions that would otherwise remain transient in the dilute cytosol. In mammalian selective autophagy, receptors such as SQSTM1, NBR1, OPTN, and TAX1BP1 therefore function not only as cargo receptors but also as multivalent organizers that increase the effective binding strength between cargo, MAP1LC3‐family proteins, and upstream scaffold factors such as RB1CC1.

This reaction‐field model is further refined by threshold‐dependent licensing mechanisms. In mammalian aggrephagy, TAX1BP1 is not a constitutive component of SQSTM1‐NBR1 condensates under basal conditions, but is recruited only when local ubiquitin load reaches a critical threshold [80]. Once recruited, TAX1BP1 promotes local assembly of TBK1‐, RB1CC1‐, and ATG9‐positive machinery, thereby converting a cargo‐collecting condensate into a phagophore‐initiation hub [80]. This threshold‐dependent licensing mechanism provides a direct mechanistic bridge between condensate organization and degradative outcome, indicating that condensates are not intrinsically degradation‐competent but instead undergo a functional state transition once cargo density and receptor occupancy surpass a critical threshold.

Experimental observations in mammalian systems support this notion. SQSTM1‐positive condensates recruit both RB1CC1 and MAP1LC3‐family proteins, creating a localized environment in which phagophore membranes can be nucleated and expanded directly at the cargo surface [19, 20, 79]. Similarly, stress granules and other RNA‐protein condensates transiently associate with autophagy factors, suggesting that these assemblies function as intermediate processing hubs rather than passive endpoints.

This organizational logic extends beyond cargo collection to later stages of autophagosome maturation. During autophagosome‐lysosome fusion, the soluble N‐ethylmaleimide‐sensitive factor attachment protein receptor (SNARE) protein SNAP29 (synaptosome‐associated protein 29) undergoes LLPS to form highly dynamic condensates that function as local assembly platforms for the STX17 (syntaxin 17)‐SNAP29‐VAMP8 (vesicle‐associated membrane protein 8) fusion machinery [84]. FRAP and live‐cell imaging analyses show rapid internal molecular exchange and frequent fusion behavior, consistent with a liquid‐like scaffold that promotes efficient SNARE complex assembly [84]. SYVN1/HRD1 (synoviolin 1) negatively regulates this process by directly binding SNAP29 and suppressing its condensation, thereby restraining autophagosome‐lysosome fusion under basal conditions [84]. These findings extend the reaction‐field concept beyond cargo processing and demonstrate that phase separation also spatially coordinates terminal membrane fusion steps.

The interface between condensates and membranes provides an additional model of organization. Productive engulfment is likely to depend not only on receptor recruitment but also on the combined effects of condensate deformability, membrane curvature, receptor density, MAP1LC3‐LIR interactions, local lipid composition, and condensate‐membrane interfacial compatibility [47, 48]. Within the proposed membrane‐wetting model, favorable interfacial coupling is expected to promote membrane spreading and productive enclosure, whereas unfavorable conditions may instead result in partial wrapping, piecemeal sequestration, or stalled phagophore association.

A broader interfacial principle is also evident outside canonical autophagy. Membrane‐bound organelles establish highly selective interfaces with biomolecular condensates, as illustrated by the presynaptic active zone, where synaptic vesicles selectively localize to the surface of condensates formed by RIMS1/RIM (regulating synaptic membrane exocytosis 1), RIMBP (RIMS binding protein), and glutamate (E), leucine (L), lysine (K), and serine (S)‐rich protein/ELKS rather than partitioning into their interior, whereas SYN (synapsin) droplets exhibit co‐phase separation with synaptic vesicles [85]. These observations suggest that condensate‐membrane interactions are governed by general interfacial compatibility rules that extend beyond autophagic engulfment and may broadly organize vesicle tethering, membrane trafficking, and organelle positioning.

This organizational logic positions condensates as self‐organizing reaction fields that integrate cargo processing, machinery assembly, threshold‐dependent licensing, and membrane dynamics. In this way, mesoscale condensate architecture feeds into subsequent fate decisions by providing the spatial context through which material properties are translated into degradative outcomes.

## 4 Fate Layer: From Condensate State to Degradation Outcome

The principles described above ultimately manifest at the level of clearance outcome. Rather than following a uniform degradative route, condensates in mammalian cells can progress toward efficient turnover, delayed yet recoverable clearance, or persistent escape states. This fate layer reflects how material properties, receptor accessibility, and membrane compatibility are integrated into productive or failed autophagic engagement.

### 4.1 Dynamic Condensates and Efficient Autophagic Clearance

Condensates that remain highly dynamic are generally the most amenable to autophagic clearance. Their liquid‐like or weakly viscoelastic properties permit rapid internal molecular exchange, maintain accessible interaction interfaces, and allow autophagy receptors to continuously sample and engage cargo molecules.

Representative examples include SQSTM1‐positive ubiquitin condensates and NCOA4‐ferritin assemblies. These assemblies display high molecular mobility and remain structurally permissive for recruitment of NBR1, OPTN, and MAP1LC3‐associated factors [19, 20, 79]. Because ubiquitin signals remain exposed and SQSTM1 oligomers are dynamically rearranged, RB1CC1 is recruited through its claw domain, enabling phagophore nucleation directly at the condensate surface [64]. In this state, the condensate functions as a degradation‐competent cargo platform that couples cargo clustering to local membrane biogenesis.

The physical deformability of these droplets is equally important. Their relatively compliant surface facilitates membrane spreading and progressive wrapping by the growing phagophore. Rather than resisting enclosure, these condensates can be partially deformed and engulfed as the membrane expands, which likely contributes to efficient lysosomal delivery. This is consistent with wetting‐based models in which low interfacial tension and preserved deformability favor productive membrane enclosure [48].

These studies define a degradable state in which fluidity, structural accessibility, and membrane compatibility are jointly preserved. In this situation, condensates remain fully competent for receptor engagement, phagophore nucleation, and subsequent lysosomal clearance.

### 4.2 Maturing Condensates and Delayed Clearance

As condensates mature, degradability often declines before complete solidification occurs. This intermediate state is important because it represents the functional decision boundary between productive turnover and pathological persistence.

Stress granules provide one of the clearest mammalian examples. Under acute oxidative or heat stress, granules assembled by G3BP1, FUS, and TARDBP remain highly dynamic and can be resolved after stress withdrawal, in part through autophagic pathways [26, 27, 28]. Under sustained stress conditions, however, internal molecular exchange progressively slows, scaffold entanglement increases, and intermolecular crosslinking becomes more pronounced. Although autophagy receptors can still be recruited to the granule surface, clearance becomes delayed and frequently incomplete.

This state is best defined by partial accessibility rather than complete resistance. Ubiquitin chains and receptor‐binding motifs remain at least partly exposed, but their accessibility is progressively reduced. Receptor turnover slows, and assembly of SQSTM1‐ or OPTN‐dependent scaffold complexes becomes less efficient. In parallel, the condensate surface becomes less compatible with phagophore spreading, reducing the efficiency of membrane wrapping and enclosure.

Functionally, these assemblies often undergo repeated cycles of recognition without productive degradation, manifesting as persistent MAP1LC3‐positive structures, incomplete engulfment, or prolonged cytoplasmic retention. This intermediate condition therefore represents not merely a transient physical state but a biologically meaningful checkpoint at which condensate fate is actively determined.

From a physical perspective, this decision boundary reflects progressive changes in both avidity and wetting compatibility. Condensates in weakly viscoelastic or gel‐like states retain sufficient receptor accessibility and surface deformability to support productive phagophore engagement. Continued hardening, however, reduces molecular exchange and weakens favorable condensate‐membrane interactions, thereby shifting the system toward delayed clearance and increasing the probability of eventual autophagic escape.

### 4.3 Pathological Solidification and Autophagic Escape

At the far end of the clearance spectrum, condensates undergo pathological hardening into highly stable solid‐like inclusions that become largely refractory to autophagic degradation. This transition is particularly evident in mammalian models of neurodegeneration.

Mutant FUS and TARDBP condensates provide well‐established examples. Disease‐associated variants accelerate the transition from liquid droplets to rigid inclusions enriched in β‐sheet interactions and fibrillar architectures [26, 28]. Similarly, MAPT/tau condensates progressively mature into insoluble aggregates that seed neurofibrillary pathology. A related mechanism involves “nested phase separation,” in which MAPT/tau undergoes a secondary fibrillar transition within pre‐existing receptor condensates, further accelerating the shift from delayed turnover to persistent escape [86]. Once this transition occurs, autophagic accessibility declines sharply.

Several mechanisms likely converge to produce this escape state. Dense molecular packing restricts access to ubiquitin signals and receptor‐docking interfaces, thereby limiting recruitment of SQSTM1, OPTN, and NBR1. At the same time, the dynamic scaffold behavior required for productive RB1CC1 recruitment is lost. In parallel, the rigid condensate surface becomes poorly compatible with membrane deformation, wetting, and progressive phagophore enclosure.

In neuronal systems, this frequently results in stalled autophagic events in which membranes partially associate with aggregates but fail to complete engulfment. Such abortive structures are observed around persistent TARDBP and MAPT/tau inclusions, indicating that molecular recognition can still occur even when productive degradation fails [87, 88]. Once established, these solidified assemblies further amplify proteotoxic stress by sequestering RNA‐binding proteins, chaperones, and signaling molecules, thereby promoting a feed‐forward cycle of persistence and toxicity. These observations support a state‐transition model in which condensate solidification marks the shift from delayed turnover to true autophagic escape.

Viewed as a whole, these three conditions define a state‐dependent decision framework for condensate fate in mammalian cells. Rather than being dictated solely by cargo identity, degradability emerges from the interplay among material state, mesoscale organization, receptor accessibility, and membrane compatibility. This framework provides a mechanistic basis for understanding how subtle shifts in condensate dynamics progressively tip the balance from physiological turnover toward disease‐associated persistence.

## 5 Disease‐Context Rewiring of Degradability Thresholds

The relationship between condensate state and autophagic fate is not static but is extensively rewired across pathophysiological contexts. Rather than invoking entirely distinct mechanisms, disease‐associated states more commonly reshape the thresholds that separate efficient clearance, delayed turnover, and autophagic escape. Viewed from this perspective, pathological conditions do not simply generate different classes of condensates; instead, they redefine the physical and biological conditions under which condensates remain degradable. Disease‐associated stress, metabolic adaptation, inflammatory signaling, mechanical remodeling, and aging collectively reshape condensate material properties, receptor accessibility, membrane compatibility, and lysosomal competence, thereby redistributing condensates across a continuum ranging from productive autophagic turnover to pathological persistence. Importantly, different diseases preferentially perturb distinct layers of this framework. Whereas neurodegeneration primarily drives progressive condensate hardening, cancer maintains metastable signaling condensates that support adaptive survival, and chronic inflammatory, fibrotic, and degenerative disorders reprogram condensate fate through disease‐specific alterations in organelle homeostasis, lipid metabolism, transcriptional regulation, or RNA processing. Disease therefore represents not merely abnormal condensate formation, but context‐dependent rewiring of condensate degradability itself (Table 4).

**TABLE 4:** Disease‐ and pathology‐associated shifts in condensate degradability thresholds.

Disease context	Representative condensates/molecules	Rewiring mechanism	Effect on autophagic fate	Pathological consequence	Refs
Neurodegeneration	FUS, TARDBP, MAPT/tau, SQSTM1	Liquid‐to‐gel/solid transition; reduced inner fluidity; impaired receptor accessibility	Delayed clearance → autophagic escape	Persistent inclusions, proteotoxic stress, neuronal degeneration	[87, 88, 89, 90, 91]
ALS‐FTD‐MSP spectrum	FUS, TARDBP, TIA1, mutant SQSTM1	Accelerated stress granule maturation; defective SQSTM1‐dependent clearance	Persistent stress granules and stalled turnover	Myodegeneration, neurodegeneration	[87, 88]
Alzheimer disease	MAPT/tau condensates	Progressive hardening and fibrillization	Reduced membrane compatibility and incomplete engulfment	Neurofibrillary pathology	[86, 89]
Mitophagy‐associated neurodegeneration	C19orf12, BNIP3	Defective mitochondrial‐surface condensate organization	Impaired mitophagy initiation	Mitochondrial dysfunction and neurodegeneration	[91]
Cancer	SQSTM1‐KEAP1‐NFE2L2, TFEB condensates	Maintenance of metastable dynamic condensates	Efficient adaptive autophagy	Tumor survival, therapy resistance	[62]
Aging and chronic proteotoxic stress	Stress granules, SQSTM1 bodies, MAPT/tau	Progressive threshold drift toward hardening	Reduced degradability across multiple condensate classes	Proteostasis collapse	[59, 61, 86]
Liver injury	Persistent SQSTM1‐KEAP1 bodies	Failed clearance with sustained signaling activity	Undegraded signaling condensates	NFE2L2 hyperactivation, hepatomegaly	[61]
Fibrosis	NCOA4‐ferritin condensates	Stress‐driven ferritinophagy rewiring	Excessive selective autophagy/ferroptotic injury	Fibrotic remodeling	[42, 43, 93]
Atherosclerosis	BRD4 and MED1 transcriptional condensates	Defective TFEB‐dependent clearance	Persistent inflammatory signaling condensates	Chronic inflammation	[94]
Vascular degeneration	PRDX1	Oxidative stress‐induced pathological phase transition	Altered stress buffering and autophagic rewiring	Ferroptosis and tissue degeneration	[95]

Abbreviations: FTD, frontotemporal dementia; MED1, mediator complex subunit 1; MSP, multisystem proteinopathy; TIA1, TIA1 cytotoxic granule‐associated RNA‐binding protein.

### 5.1 Neurodegeneration: Pathological Hardening and Autophagic Escape

Neurodegeneration provides the clearest example of disease‐associated threshold rewiring. Proteins such as FUS, TARDBP/TDP‐43, MAPT/tau, and SQSTM1 progressively lose fluidity and evolve from dynamic liquid‐like condensates into persistent gel‐like or solid assemblies, thereby reducing receptor accessibility and membrane compatibility [87, 88, 89, 90, 91]. Rather than representing passive end‐products of proteotoxic stress, these hardened condensates increasingly function as pathological reaction fields that promote aberrant protein–protein interactions, fibrillization, and defective quality control.

Recent studies further demonstrate that condensate maturation actively accelerates disease progression. For example, the autophagy adaptor RNF216/TRIAD3A (ring finger protein 216) promotes MAPT/tau fibrillation through nested phase separation, suggesting that autophagy‐associated condensates themselves can become catalytic environments that facilitate amyloid maturation rather than simply serving as degradation substrates [86]. Likewise, amyotrophic lateral sclerosis (ALS)‐associated *SQSTM1* mutations generate phase‐separated droplets with reduced internal fluidity, indicating that disease‐associated mutations directly impair condensate dynamics before irreversible aggregation occurs [88]. Consistent with this model, pharmacological stabilization of the liquid state by myricetin delays tau phase separation while simultaneously enhancing ATG5‐dependent autophagy, thereby suppressing tau toxicity [89].

Failure of condensate clearance reflects more than impaired receptor recognition. Progressive condensate hardening reduces molecular permeability, limits accessibility of MAP1LC3‐interacting receptors, decreases condensate deformability, and weakens membrane engulfment efficiency. These changes shift condensates beyond the degradable region of the material‐state continuum, resulting in persistent autophagic intermediates and incomplete cargo sequestration. Additional defects in mitochondrial quality control further amplify this process. Phase separation of C19orf12 (chromosome 19 open reading frame 12), for example, regulates BNIP3 (BCL2 interacting protein 3)‐dependent mitochondrial protein quality control and neuronal mitophagy, illustrating how condensate dysfunction extends beyond protein aggregation to organelle homeostasis [91].

Collectively, neurodegeneration therefore represents a progressive leftward shift of the condensate degradability landscape, in which mutations, aging, and chronic proteotoxic stress jointly accelerate liquid‐to‐solid transitions while simultaneously reducing the efficiency of receptor recruitment, membrane engagement, and lysosomal clearance.

### 5.2 Cancer: Preservation of Adaptive Metastable Condensates

Cancer rewires the degradability landscape in a fundamentally different manner. Rather than driving irreversible condensate hardening, tumor cells frequently maintain condensates in metastable and partially dynamic states that maximize signaling plasticity, metabolic adaptation, and stress tolerance. Under nutrient limitation, oxidative stress, hypoxia, and proteotoxic burden, condensate‐mediated organization becomes an adaptive mechanism that coordinates selective autophagy with cellular survival.

Persistent SQSTM1‐KEAP1 condensates provide one representative example. Under conditions of impaired autophagic turnover, phase‐separated SQSTM1 bodies retain KEAP1, thereby sustaining NFE2L2 activation and reinforcing antioxidant defense pathways [61, 62]. Rather than functioning solely as degradable cargo, these condensates become dynamic signaling platforms that buffer oxidative stress and maintain redox homeostasis. Similarly, phase‐separated TFEB condensates regulate transcriptional activity of the autophagy–lysosome pathway through their material properties, thereby coordinating lysosomal biogenesis, autophagic capacity, and metabolic adaptation [53].

Emerging evidence further suggests that oncogenic condensates constitute an integrated regulatory network supporting malignant progression [92]. Instead of allowing condensates to mature into rigid inclusions, cancer cells preserve sufficient condensate liquidity to permit continuous molecular exchange, rapid signaling reconfiguration, and efficient recycling of damaged macromolecules. This adaptive state enables tumor cells to balance anabolic growth with selective degradation, thereby enhancing resistance to metabolic stress and therapeutic intervention.

Thus, cancer represents a distinct context for condensate degradability. Instead of promoting irreversible condensate hardening, tumor cells often preserve condensates in a metastable state that remains compatible with dynamic turnover and adaptive autophagy. Consequently, therapeutic strategies that enhance condensate clearance may be beneficial in neurodegenerative disorders but could also support tumor cell adaptation under stress.

### 5.3 Fibrotic Remodeling: Rewiring Lipid‐Associated Condensate Degradation

Fibrotic remodeling represents a distinct mode of disease‐specific rewiring in which condensate dynamics intersect with organelle metabolism rather than classical proteotoxicity. Activated fibroblasts and hepatic stellate cells undergo profound metabolic remodeling characterized by altered lipid‐droplet turnover, mitochondrial adaptation, and persistent extracellular matrix production, processes that are increasingly linked to condensate‐dependent regulation of selective autophagy.

A recent study demonstrates that the garlic‐derived metabolite diallyl trisulfide (DAT) regulates RAB18 phase separation, thereby suppressing lipophagy and inducing cuproptosis in activated hepatic stellate cells to attenuate liver fibrosis [93]. The study combines human LX‐2 hepatic stellate cells, primary hepatic stellate cells, mouse models of liver fibrosis, and human fibrotic liver tissues, providing evidence across multiple biological scales that condensate remodeling directly regulates disease progression. Mechanistically, DAT promotes RAB18 condensation at lipid droplets and mitochondria‐associated membranes, alters lipid utilization, inhibits excessive lipophagy, and ultimately sensitizes activated stellate cells to copper‐dependent cell death [93]. Rather than functioning solely as structural assemblies, RAB18 condensates serve as dynamic metabolic platforms that coordinate organelle communication, lipid mobilization, and selective autophagy.

These findings suggest that condensate degradability in fibrosis is governed primarily by metabolic organelle organization rather than pathological protein aggregation. Persistent RAB18‐dependent condensates support metabolic adaptation and fibroblast survival, whereas remodeling condensate material properties redirects lipid metabolism toward cuproptosis, thereby limiting fibrotic progression.

### 5.4 Inflammatory Disease: Persistence of Signaling Condensates

Inflammatory diseases illustrate a distinct mode of condensate rewiring in which defective autophagic turnover prolongs signaling rather than permitting passive protein accumulation. Under physiological conditions, stress‐responsive condensates assemble transiently to coordinate innate immune signaling and are subsequently dismantled through selective autophagy. Chronic inflammatory stress, however, shifts these assemblies toward persistent signaling platforms that continue to recruit downstream effectors despite impaired degradative turnover.

A representative example is provided by SQSTM1‐dependent P‐bodies, which form under prolonged endotoxin stress and other conditions that enhance SQSTM1 condensation [60]. Experimental studies in macrophage‐based inflammatory models, combined with proteomic analyses and live‐cell imaging, demonstrate that phase‐separated SQSTM1 condensates recruit the RNA helicase DDX6, thereby generating enlarged SQSTM1‐dependent P‐bodies that subsequently recruit PYCARD/ASC and promote NLRP3 inflammasome activation [60].

Inflammatory condensates are also regulated at the transcriptional level. In experimental atherosclerosis, curcumin activates autophagy through the TFEB‐P300‐BRD4 (bromodomain containing 4) signaling axis, thereby restoring lysosomal function while suppressing inflammatory gene expression [94]. Evidence from *ApoE^−/−^* mice, vascular endothelial cells, and macrophage models of atherogenesis further demonstrates that activation of TFEB‐dependent lysosomal programs reduces plaque formation and inflammatory injury [94]. Rather than acting solely through bulk autophagy, this pathway remodels transcriptional condensates that coordinate lysosomal biogenesis and inflammatory signaling.

Together, these findings indicate that inflammatory diseases are characterized by persistent signaling competence rather than irreversible condensate hardening. In this context, defective condensate turnover sustains innate immune signaling while condensates remain sufficiently dynamic to recruit downstream effectors, representing a distinct mode of condensate degradability from that observed in neurodegenerative proteinopathies.

### 5.5 Vascular Degeneration: Condensate Homeostasis and Redox Adaptation

Vascular degeneration links condensate dynamics to endothelial homeostasis, oxidative stress, and ferroptotic susceptibility. A recent study demonstrates that KIF11 (kinesin family member 11) suppresses phosphorylation‐dependent PRDX1 (peroxiredoxin 1) phase separation, thereby preserving antioxidant activity and protecting retinal endothelial cells against ferroptosis in familial exudative vitreoretinopathy [95]. Evidence from human retinal microvascular endothelial cells, disease‐associated KIF11 variants, and mouse models of retinal vascular degeneration further demonstrates that excessive PRDX1 condensation impairs peroxide detoxification, enhances lipid peroxidation, and promotes endothelial ferroptosis [95]. Restoration of condensate dynamics rescues vascular integrity, indicating that maintenance of condensate liquidity is required for endothelial homeostasis.

Unlike neurodegenerative proteinopathies, where condensate persistence primarily promotes pathological aggregation, vascular degeneration arises from impaired redox‐active condensates that lose sufficient molecular exchange to sustain antioxidant function. Consequently, disease progression reflects defective condensate homeostasis that compromises redox adaptation before irreversible structural damage occurs.

### 5.6 Ferroptotic and Metabolic Diseases: Coupling Transcriptional Condensates With Selective Autophagy

Ferroptotic disorders provide another example in which condensate rewiring operates across multiple biological scales. Ferritinophagy normally depends on NCOA4‐mediated ferritin condensates, which deliver ferritin to lysosomes for degradation and regulate intracellular iron homeostasis [38, 39, 40]. Moderate ferritinophagy supports physiological iron recycling, whereas excessive ferritin degradation increases the labile iron pool and sensitizes cells to lipid peroxidation and ferroptosis [42, 43].

Experimental studies in chronic unpredictable stress mouse models, cultured neuronal cells, chromatin occupancy analyses, and phase‐separation assays demonstrate that BRD2 undergoes phase separation to activate super‐enhancer‐driven ATG7 transcription, thereby promoting ferritinophagy in experimental depression [96]. Disruption of BRD2 condensates reduces ATG7 expression, attenuates ferritinophagy, and alleviates depression‐like behavioral phenotypes [96]. These findings establish a hierarchical regulatory circuit in which nuclear transcriptional condensates regulate the degradability of cytoplasmic ferritin condensates through transcriptional control of the autophagy machinery.

Additional evidence comes from mechanically induced ferroptosis, where activation of the NCOA4‐FTH1 axis couples mechanical stress to ferritinophagy and ferroptotic susceptibility [42], and from pulmonary fibrosis models, in which NCOA4‐mediated ferritin condensates promote ferritin‐containing extracellular vesicle release and fibrotic remodeling [43]. Collectively, these studies indicate that ferroptotic disorders arise from coordinated regulation of transcriptional condensates, ferritin condensates, and selective autophagy rather than from defects in ferritin degradation alone.

### 5.7 Tissue‐Specific Degenerative Disorders: Expanding Condensate Degradability Beyond Proteostasis

Tissue‐specific disorders further broaden the biological scope of condensate degradability by demonstrating that condensate‐dependent autophagy regulates RNA metabolism, mitochondrial quality control, and developmental homeostasis in addition to protein aggregation.

One representative example is provided by the WW domain‐containing adaptor with coiled‐coil (WAC). Experimental studies using cellular models, transcriptomic analyses, phase‐separation assays, and mouse validation demonstrate that WAC forms nuclear condensates that regulate mitophagy through alternative RNA splicing [97]. WAC condensates coordinate alternative splicing of mitophagy‐associated genes, thereby linking nuclear condensate organization to mitochondrial quality control [97]. Unlike canonical cargo receptors that act directly on damaged mitochondria, WAC regulates mitophagy at the level of gene expression, illustrating that condensate degradability can be specified before cargo recognition occurs.

A conceptually related mechanism is observed in reproductive biology. Evidence from human trophoblast cell models, patient tissues, and mouse pregnancy models demonstrates that Lnc‐HZ14 promotes LLPS‐mediated SPHK1 (sphingosine kinase 1) aggrephagy, thereby suppressing trophoblast proliferation in unexplained recurrent miscarriage [98]. RNA‐driven condensates selectively recruit SPHK1 into autophagic degradation pathways, ultimately impairing placental development [98]. These findings indicate that long noncoding RNAs actively regulate condensate degradability by directing the selective autophagic turnover of signaling proteins.

Together with mitochondria‐dependent phase separation driving pathological protein accumulation in age‐related macular degeneration [63], these examples expand condensate degradability beyond classical proteostasis to include regulation of mitochondrial quality control, RNA processing, tissue development, and cell‐fate determination.

### 5.8 Aging: Systems‐Level Drift of Condensate Degradability

Unlike individual diseases, aging represents a systems‐level perturbation that progressively reshapes virtually every component of the condensate degradability framework. Declining chaperone buffering, impaired lysosomal function, reduced ATP availability, mitochondrial dysfunction, and cumulative oxidative damage collectively narrow the degradable region of the condensate‐state landscape [50, 59, 61, 86].

Rather than affecting a single condensate class, aging simultaneously promotes stress granule maturation, persistent SQSTM1 bodies, STAU1 condensates, pathological MAPT/tau assemblies, and defective mitochondrial quality control [59, 61, 86]. As lysosomal competence declines, condensates remain exposed to prolonged stress histories, progressively accumulating intermolecular crosslinks that further reduce receptor accessibility and membrane compatibility. Consequently, impaired degradation accelerates condensate hardening, whereas condensate hardening further overloads autophagic capacity, establishing a self‐reinforcing cycle that progressively drives cells toward pathological persistence.

## 6 Therapeutic Engineering and Translational Perspectives

The growing mechanistic understanding of the phase separation–autophagy axis is creating new opportunities for therapeutic intervention. Rather than simply enhancing or suppressing autophagy, emerging strategies increasingly aim to engineer condensate degradability by modulating the determinants that govern selective cargo recognition and autophagic processing. Within the framework proposed here, therapeutic intervention can target multiple organizational levels, including restoration of receptor accessibility, preservation of favorable material properties, optimization of condensate–membrane engagement, enhancement of lysosomal competence, and selective redirection of condensate fate. This perspective shifts therapeutic design from global regulation of autophagic flux toward rational engineering of condensate behavior. Although these approaches remain at an early stage, they collectively define multiple mechanistic entry points for therapeutic manipulation of condensate degradability.

### 6.1 Engineering Scaffold‐Dependent Condensate Recognition

One strategy is to engineer scaffold‐driven condensate recognition. Selective autophagy receptors such as SQSTM1 organize ubiquitinated cargo into multivalent condensates that function as mesoscale reaction fields for receptor recruitment and autophagosome initiation. Experimental studies in breast cancer cells demonstrate that the NanoTACOrg platform mimics SQSTM1‐mediated scaffold organization to achieve programmable sequestration of intracellular organelles [99]. Rather than targeting individual proteins, NanoTACOrg reconstructs the multivalent architecture of selective autophagy, enabling selective clustering and lysosomal degradation of mitochondria, endoplasmic reticulum, and Golgi membranes. These findings demonstrate that the organizational principles of endogenous autophagy receptors can be translated into synthetic degradation platforms.

### 6.2 Preserving Condensate Material Properties

A complementary strategy is to preserve or remodel condensate material properties before irreversible hardening occurs. Progressive transitions from liquid‐like droplets to gel‐like or solid assemblies reduce receptor accessibility, molecular exchange, and membrane compatibility, thereby shifting condensates beyond the degradable regime. Although direct pharmacological reversal of pathological hardening remains limited, condensate liquidity, interfacial tension, and molecular permeability are emerging as therapeutically tractable properties. Experimental studies in tumor models demonstrate that ruthenium(II)‐based lipid mimetics induce membrane phase separation, reorganize membrane‐associated condensates, enhance ferritinophagy‐dependent iron release, and activate an autophagy–ferroptosis cascade that potentiates photoimmunotherapy [100]. Rather than directly targeting ferritin degradation, this approach reprograms membrane organization to redirect downstream autophagic outcomes.

### 6.3 Reprogramming Endogenous Condensate‐Regulatory Circuits

Therapeutic intervention may also exploit endogenous pathways that regulate condensate assembly and degradation. Experimental studies using gastric cancer cells, patient‐derived specimens, and mouse metastasis models demonstrate that the FDA‐approved antiviral drug lopinavir restores the c*ircSPECC1*‐ATG4B regulatory circuit, which controls autophagy through RNA‐dependent condensate remodeling [101]. Restoration of *circSPECC1*‐dependent phase separation of ATG4B promotes anoikis and suppresses metastatic dissemination, illustrating that endogenous condensate‐regulatory circuits can be therapeutically reprogrammed without constructing synthetic condensates [101].

### 6.4 Optimizing Condensate‐Membrane Engagement and Lysosomal Competence

An additional opportunity is to optimize condensate‐membrane engagement. Productive engulfment depends not only on receptor recruitment but also on condensate deformability, interfacial tension, membrane wetting, lipid composition, and phagophore curvature [47, 48, 83]. Although direct therapeutic modulation of these interactions has not yet been achieved, improving condensate wrapping, membrane deformation, or receptor avidity may enhance selective autophagy without globally stimulating autophagic activity. Likewise, restoration of lysosomal competence, for example, through TFEB‐dependent activation of the autophagy–lysosome pathway, may expand the range of condensates that remain degradable by increasing cellular degradative capacity [52, 53].

### 6.5 Context‐Dependent Therapeutic Intervention

Therapeutic manipulation of condensate degradability is inherently context‐dependent. In neurodegenerative diseases, restoring condensate liquidity or enhancing selective autophagic clearance is expected to facilitate elimination of persistent protein condensates and preserve proteostasis. In contrast, many cancers maintain metastable condensates and adaptive autophagy to support mitochondrial quality control, metabolic flexibility, and resistance to oxidative stress [53, 61, 62, 92]. Consequently, indiscriminate enhancement of autophagy or condensate degradation may inadvertently promote tumor survival or therapeutic resistance. Similar considerations apply to inflammatory diseases, where excessive elimination of signaling condensates may compromise physiological innate immune responses, whereas insufficient clearance sustains inflammasome activation [60, 94]. Therapeutic strategies should therefore redirect condensate fate according to disease context rather than globally activating or inhibiting autophagy.

Future therapeutic development is therefore likely to focus on precision engineering of condensate degradability, integrating receptor accessibility, condensate material properties, membrane engagement, and lysosomal competence to selectively remodel autophagic outcome according to disease context. Such a framework provides a mechanistic basis for translating condensate biology into context‐dependent therapeutic strategies.

## 7 Conclusion and Future Perspectives

This review highlights a framework in which condensate fate is governed by the interplay among material dynamics, spatial organization, and membrane coupling. A central challenge for the field now lies not in further cataloging individual examples, but in defining generalizable and experimentally testable principles that predict whether a condensate undergoes efficient clearance, delayed turnover, or persistent escape across distinct cellular contexts.

Several major unresolved questions now define the next phase of the field. First, what quantitative thresholds distinguish degradable, delay‐prone, and escape‐associated condensate states? Although parameters such as internal exchange kinetics, deformability, receptor occupancy, and condensate‐membrane interfacial compatibility have emerged as candidate predictors, the relative weighting and predictive value of these variables remain unclear. Establishing experimentally validated fate maps that integrate live‐cell imaging, FRAP‐based kinetics, cryo‐electron tomography, and computational modeling will therefore be a major priority.

A second unresolved issue concerns context specificity. The thresholds governing condensate fate are unlikely to be universal and may vary substantially across cell types, organelles, and stress conditions. Long‐lived cells such as neurons may be particularly vulnerable to small shifts in condensate persistence, whereas rapidly proliferating cells may actively maintain condensates within a degradable adaptive regime. Defining how these thresholds are rewired across neurodegeneration, cancer, aging, and tissue injury remains essential for understanding disease‐selective vulnerability.

Another major open question concerns the general applicability of the proposed membrane‐wetting model across distinct selective autophagy pathways. Rather than representing a universal rule, membrane wetting should currently be regarded as a working model whose contribution to condensate engulfment is likely to depend on multiple factors, including receptor density, MAP1LC3‐LIR interactions, membrane curvature, local lipid composition, condensate deformability, and lysosomal competence. Defining how these variables collectively influence complete engulfment, partial wrapping, piecemeal degradation, or stalled phagophore association remains an important direction for future investigation.

The translational trajectory of the field is increasingly centered on state‐guided intervention strategies. Rather than globally enhancing or suppressing autophagy, future therapeutic approaches should aim to selectively remodel pathological condensates while preserving the physiological liquid‐like condensates that are essential for normal cellular organization. Achieving this selectivity represents one of the major translational challenges in the field. Potential strategies include selectively modulating condensate material properties, receptor coupling, post‐translational modifications, molecular chaperones, or condensate–membrane interactions to restore degradability without compromising physiological condensate dynamics.

In parallel, engineered phase‐separation systems are beginning to serve as analytical tools for interrogating autophagy in living cells. Representative examples include the SPARK‐based PINK1 condensate biosensor and lysosome‐targeted phase‐sensitive fluorescent probes [102, 103], which provide real‐time readouts of signaling activation, lysosomal remodeling, and mitophagy dynamics, creating new opportunities to quantitatively monitor fate transitions in situ. Future integration of synthetic condensate engineering, quantitative biophysical measurements, and disease models should further facilitate mechanistic validation of condensate fate and therapeutic intervention strategies.

Ultimately, the next major advance will be the establishment of predictive and experimentally testable models that integrate condensate material properties with receptor engagement, membrane interactions, and cellular clearance capacity to forecast autophagic outcomes across physiological and pathological settings. Such models should enable discrimination between physiological condensates and pathological assemblies, providing a conceptual foundation for condensate‐selective therapeutic strategies that restore degradability while preserving normal phase behavior. Together, this framework provides a unifying basis for both mechanistic discovery and translational development in mammalian autophagy biology.

## Author Contributions

**Yu Sun**: Writing – review and editing, conceptualization. **Junnan Jiang**: writing – review and editing, conceptualization. **Daniel J. Klionsky**: writing – review and editing. **Ningning Lv**: writing – review and editing, conceptualization. **Yuanqiang Lin**: writing – original draft. **Daolin Tang**: writing – review and editing, conceptualization. **Rui Kang**: writing – review and editing. **Enyong Dai**: writing – original draft. **Mingran Dai**: writing – original draft.

## Funding

This work was supported by the Natural Science Foundation of the Jilin Provincial Department of Science and Technology (General Project; Project No. 20210101331JC).

## Conflicts of Interest

The authors declare no conflicts of interest.

## Data Availability

This article is a review of previously published studies. No new data were generated or analyzed during the current study.
